# An Antibiotic Nanobomb Constructed from pH‐Responsive Chemical Bonds in Metal‐Phenolic Network Nanoparticles for Biofilm Eradication and Corneal Ulcer Healing

**DOI:** 10.1002/advs.202309086

**Published:** 2024-03-15

**Authors:** Qiang Gao, Xiaoying Chu, Jie Yang, Yishun Guo, Hanwen Guo, Siyuan Qian, Ying‐Wei Yang, Bailiang Wang

**Affiliations:** ^1^ National Engineering Research Center of Ophthalmology and Optometry Eye Hospital Wenzhou Medical University Wenzhou 325000 P. R. China; ^2^ State Key Laboratory of Ophthalmology, Optometry and Visual Science Wenzhou Medical University Wenzhou 325027 P. R. China; ^3^ School of Life Sciences Jilin University 2699 Qianjin Street Changchun 130012 P. R. China; ^4^ College of Chemistry Jilin University 2699 Qianjin Street Changchun 130012 P. R. China; ^5^ NMPA Key Laboratory for Clinical Research and Evaluation of Medical Devices and Drug for Ophthalmic Diseases Wenzhou 325027 P. R. China

**Keywords:** antibacterial, corneal ulcers, metal‐phenolic network, modular assembly, pH responsiveness

## Abstract

In the treatment of refractory corneal ulcers caused by *Pseudomonas aeruginosa*, antibacterial drugs delivery faces the drawbacks of low permeability and short ocular surface retention time. Hence, novel positively‐charged modular nanoparticles (NPs) are developed to load tobramycin (TOB) through a one‐step self‐assembly method based on metal‐phenolic network and Schiff base reaction using 3,4,5‐trihydroxybenzaldehyde (THBA), ε‐poly‐ʟ‐lysine (EPL), and Cu^2+^ as matrix components. In vitro antibacterial test demonstrates that THBA‐Cu‐TOB NPs exhibit efficient instantaneous sterilization owing to the rapid pH responsiveness to bacterial infections. Notably, only 2.6 µg mL^−1^ TOP is needed to eradicate *P. aeruginosa* biofilm in the nano‐formed THBA‐Cu‐TOB owing to the greatly enhanced penetration, which is only 1.6% the concentration of free TOB (160 µg mL^−1^). In animal experiments, THBA‐Cu‐TOB NPs show significant advantages in ocular surface retention, corneal permeability, rapid sterilization, and inflammation elimination. Based on molecular biology analysis, the toll‐like receptor 4 and nuclear factor kappa B signaling pathways are greatly downregulated as well as the reduction of inflammatory cytokines secretions. Such a simple and modular strategy in constructing nano‐drug delivery platform offers a new idea for toxicity reduction, physiological barrier penetration, and intelligent drug delivery.

## Graphical abstract

Novel positively‐charged modular nanoparticles (NPs) are developed to load tobramycin (TOB) through a one‐step self‐assembly method based on metal‐phenolic network and Schiff base reaction. Like a nano‐grenade, NPs exhibit efficient instantaneous sterilization owing to the rapid pH responsiveness to bacterial infections. Such a simple and modular strategy in constructing nano‐drug delivery platforms offers a new idea for toxicity reduction, physiological barrier penetration, and intelligent drug delivery.

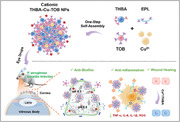

## 1 Introduction

Refractory corneal ulcers induced by *Pseudomonas aeruginosa* (*P. aeruginosa*) and related biofilm mainly lead to difficult removal of infections, severe inflammatory reactions, and corneal tissue damage. The biofilm cannot be entirely eradicated through debridement because the exopolysaccharides (EPS) matrix will penetrate the deep tissue structure, and the residue will make the biofilm recover quickly.^[^ ^1^ ^]^ Like a “bacteria nest”, the biofilm leads to increased drug resistance, repeated infections, and chronic inflammatory response.^[^ ^2^ ^]^ In combination with quorum sensing and sharing of drug resistance genes, the long‐term exposure of bacteria under sublethal antibiotic concentration will further promote the development of bacterial resistance, which needs a lethal concentration of 500–1000 times antibiotic dose compared to free bacteria.^[^ ^3^ ^]^ At present, new bactericidal strategies, including photodynamic therapy, photothermal therapy, sonodynamic therapy, and single atom nanozymes, show efficient sterilization and even biofilm damage effect.^[^ ^4^ ^]^ Nevertheless, it is still a great challenge to apply them in clinical practice for such drawbacks as external energy penetration, material toxicity, engineering transformation, *etc*.^[^ ^5^ ^]^

Tobramycin (TOB) is an aminoglycoside antibiotic that can be used to treat various bacterial infections, especially those caused by Gram‐negative bacteria, such as severe *P. aeruginosa* infections.^[^ ^6^ ^]^ However, due to the short ocular surface retention time, low permeability to corneal physiological barriers and bacterial biofilm, and potential toxicity, the therapeutic effect of free TOB drops in treating refractory corneal ulcer is greatly compromised in clinical practice. Specifically, the short retention time of eye drops and corneal barrier with unique hydrophilic‐hydrophobic alternating layers significantly reduce the bioavailability of drugs.^[^ ^7^ ^]^ Therefore, researchers have constructed a series of delivery systems for sustained drug release, including drug gels, drug‐loaded contact lenses, and nano drug delivery systems such as liposomes and chitosan NPs to improve the retention time of drugs on the ocular surface and the penetration into corneal physiological barrier.^[^ ^8^ ^]^ However, these drug delivery systems have shortcomings in drug loading, release controllability, and penetration of bacterial biofilm. Moreover, these drug delivery systems also lack the role in inflammation elimination and promoting corneal wound healing.

The inflammatory response caused by corneal infection is also a knotty problem that needs to be solved, which causes irreversible damage to the cornea and decrease light transmittance.^[^ ^9^ ^]^ Once infection happens, bacterial toxins stimulate the infected site to produce a large number of inflammatory mediators, such as inflammatory factors and chemokines, inducing inflammatory cell infiltration and oxidative stress.^[^ ^10^ ^]^ The presence of bacterial biofilm significantly enhances the resistance of bacteria to antibiotics, making it difficult to eliminate infections. In addition, the high expression of various enzymes such as phosphatase, phospholipase, toxin, lipase, and protease in the infected area leads to irreversible damage of corneal tissue and decreased light transmittance.^[^ ^11^ ^]^ As the toxic component on the outer membrane of Gram‐negative bacteria, lipopolysaccharide (LPS)^[^ ^12^ ^]^ can interact with toll‐like receptor 4 (TLR4) receptor on macrophage membrane,^[^ ^13^ ^]^ and then activate intracellular inflammatory response through nuclear factor kappa B (NF‐κB) signaling pathway,^[^ ^14^ ^]^ thereby resulting in the production of pro‐inflammatory mediators including tumor necrosis factor‐α (TNF‐α), interleukin‐6 (IL‐6), and interleukin‐1β (IL‐1β).^[^ ^15^ ^]^ Generally, anti‐inflammatory hormone drugs are used about a week after treatment with antibacterial drugs. However, the side effects of these drugs are also very obvious. For example, glucocorticoids can induce cataract and ocular hypertension, and non‐steroidal anti‐inflammatory drugs may melt cornea.^[^ ^16^ ^]^ It is worth mentioning that oxidative stress and inflammatory reactions are simultaneously activated, accompanied, interacted, and stimulated during the sterilization process. It has been proven that scavenging oxidative free radicals can effectively eliminate oxidative stress and further reduce inflammatory responses.^[^ ^4^, ^17^ ^]^ For example, polyphenols (e.g., tannic acid, epigallocatechin gallate, gallic acid, and protocatechuic aldehyde) display significant reactive oxygen species (ROS) scavenging property through H‐atom transfer, and decreasing noxious effects caused by oxidative stress. Polyphenols can also inhibit the nuclear transfer of NF‐κB by impeding the phosphorylation of kinase and interfere with the binding of active NF‐κB to impede the inflammation.^[^ ^18^ ^]^

Therefore, it is urgent to construct an antibiotic drug loading system in a convenient way to achieve high permeability, biofilm elimination, and inflammatory damage reduction in refractory corneal ulcers treatment. The infected area is generally believed to be weakly acidic in the range of 5.0‐6.5,^[^ ^19^ ^]^ which facilitates the design and construction of acid responsive nano‐drug delivery systems.^[^ ^20^ ^]^ Metal‐phenolic networks (MPNs), as a newly developed organic‐inorganic hybrid system, show conspicuous anti‐oxidation and anti‐inflammatory features and promotion of blood vessel repair ability owing to the components of polyphenols and metal ions, respectively.^[^ ^21^ ^]^ Interestingly, the network structure formed by the coordination of metal ions and phenolic hydroxyl groups in MPNs is responsive to pH, allowing the release of components in a slightly acidic environment.^[^ ^22^ ^]^ Moreover, MPNs with distinct designability are expected to be used as the modular assembly nanoplatforms to realize the combination of polyphenol‐like compounds. Therefore, it is believed that drugs such as TOB with both phenolic hydroxyl and aldehyde groups could be co‐assembled into MPNs through formation of Schiff base between aldehyde groups and amino groups. In this way, a new drug loaded MPNs is designed with positive charge characteristic, adjustable drug loading capacity, rapid pH response to bacterial infection microenvironment (pH 5.0‐‐7.0) for bacteria sterilization, inflammation elimination, and promoting corneal epithelial cell migration comprehensive effect.

The combination of positively‐charged NPs and negatively‐charged mucin in the tear layer through electrostatic interactions is beneficial to improve the ocular surface retention time and stimuli‐responsive release of drugs.^[^ ^7^, ^23^ ^]^ Similarly, positively‐charged NPs not only improve the binding and bactericidal effects by destroying the integrity of bacterial membrane via electrostatic interactions but also enhance the biofilm eradication ability based on the good permeability and retention of the modular NPs.^[^ ^24^ ^]^ Herein, we put forward a proof‐of‐concept strategy to cure *P. aeruginosa* infected corneal ulcers through modular nanomaterials construction based on the following functions: (a) improving drug retention time on the ocular surface and corneal physiological barrier penetration; (b) enhancing biofilm permeability for rapid sterilization; (c) scavenging ROS for anti‐inflammation; (d) promoting corneal epithelial cell migration and wound healing. Therefore, the pH‐responsive MPNs drug delivery system is expected to realize desirable corneal ulcer healing under the comprehensive action of sterilization, oxidative stress elimination, inflammation reduction, and corneal epithelial cell migration promotion.

## 2 Results and Discussion

### 2.1 Synthesis and Characterization of THBA‐Cu‐TOB NPs

**Scheme 1:**
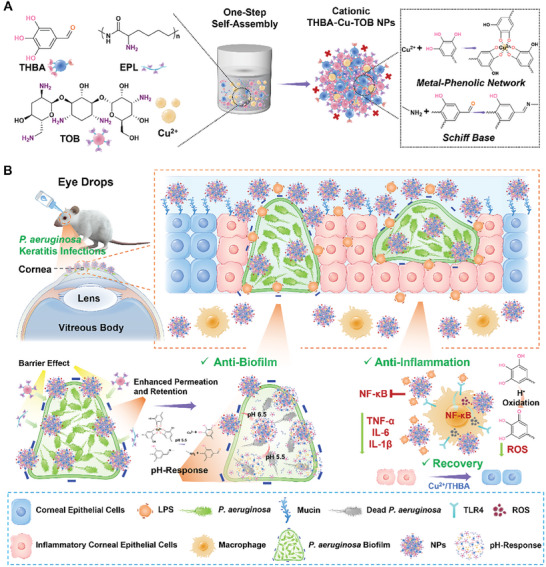
Schematic diagram of A) the fabrication of cationic THBA‐Cu‐TOB NPs and B) the application in the synergistic treatment of infectious corneal ulcer.

The modular assembled cationic THBA‐Cu‐TOB NPs consist of 3,4,5‐trihydroxybenzaldehyde (THBA), ε‐poly‐ʟ‐lysine (EPL), Cu^2+^, and TOB were synthesized in a simple, low‐cost, and one‐pot way (**Scheme** **1**A). THBA‐Cu‐TOB NPs were administered in the form of eye drops onto the surface of established corneal ulcer lesions infected by *P. aeruginosa*. The rational mechanism of corneal infection healing is illustrated as follows (Scheme 1B). First, EPL‐endowed THBA‐Cu‐TOB NPs with positively charges enhanced ocular surface adhesion for prolonged drug retention time and improved penetration into both *P. aeruginosa* biofilm and corneal physiological barrier. Notably, TOB could be rapidly released through simultaneous cleavage of MPN and Schiff base bonds in acidic bacterial infection environment for infection elimination and biofilm eradication. Besides, polyphenol component worked in both ROS scavenging and anti‐inflammatory effects. In addition, Cu^2+^ promoted the migration of human corneal epithelial cells (HCECs) for corneal wound healing.

**Figure 1:**
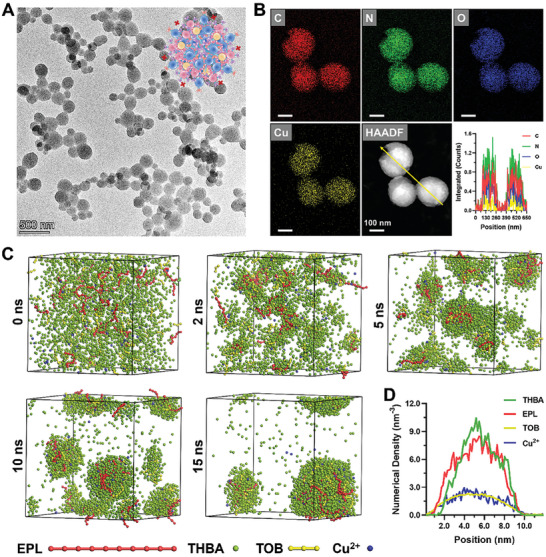
A) TEM and B) corresponding element (C, N, O, and Cu) mapping images for THBA‐Cu‐TOB NPs. Inside A, upper‐right corner: schematic representation of the morphology of NPs. Inset B: line‐scan STEM elemental distribution (yellow arrow) of THBA‐Cu‐TOB NPs. C) Coarse‐grained simulation of self‐assembling THBA‐Cu‐TOB NPs using a DPD method. Simulation box containing EPL (red), THBA (green), TOB (yellow), and Cu^2+^ (blue) in solution, and a snapshot of NPs formed spontaneously after t_CG_ = 0, 2, 5, 10, and 15 ns, respectively. *Note: the C_2_H_5_OH and H_2_O molecules are not shown*. D) Snapshot of simulated THBA‐Cu‐TOB NPs at t_CG_ = 15 ns and the density distribution function of four components in simulated NPs.

Both THBA‐Cu‐TOB and THBA‐Cu were fabricated through facile one‐step method. As shown in **Figure** **1**A,B and Figure S1 (Supporting Information), THBA‐Cu and THBA‐Cu‐TOB NPs displayed a spheroidal shape with a relatively uniform size distribution and homogeneously distributed C, N, O, and Cu elements as measured in transmission electron microscope (TEM), energy dispersive spectroscopy (EDS) elemental mapping, and high‐angle annular dark‐field imaging‐scanning TEM (HAADF‐STEM) images. In addition, element EDS line scan analysis was performed along the yellow arrow across two NPs indicating the general content of C, N, O, and Cu elements. The main component of NPs was THBA, which provides polyphenol groups and aldehyde groups to react with Cu^2+^ and the amino groups on EPL and TOB to perform metal‐polyphenol and Schiff base reaction, respectively.^[^ ^25^ ^]^

To further explore the self‐assembly of THBA‐Cu‐TOB NPs, the formation process was studied by coarse‐grained molecular simulations using a dissipative particle dynamics (DPD) method (Figure 1C). All components including EPL, THBA, TOB, and Cu^2+^ were evenly distributed in the C_2_H_5_OH/H_2_O solution. After 5 ns evolution, the components spontaneously assembled to produce a large number of small particles. At 15 ns, more large particles formed due to the mutual attraction between the small particles. The distribution curves were obtained by calculating the density distribution function of a single particle along the radius direction (Figure 1D). The density distribution function was the average occurrence frequency of each component per unit volume at a given radius. In the initial stage of nanomaterials formation, the long chain shaped EPL occupied the primary proportion of the four components and played a similar role as a “crystal seed”. The aggregation and bonding of THBA on the molecular chain further led to the aggregation and bonding of TOB and Cu^2+^. Moreover, the aggregation speed of TOB was very fast, and it also occupied the main component of nanomaterials in the initial stage. Nanoaggregates with a size of about 10 nm formed within 15 ns, indicating strong and rapid bonding between these four components. These results suggested that the formation of two pH responsive chemical bonds was very fast, effectively achieving the uniform loading of antibiotics TOB within the nanomaterials.

**Figure 2:**
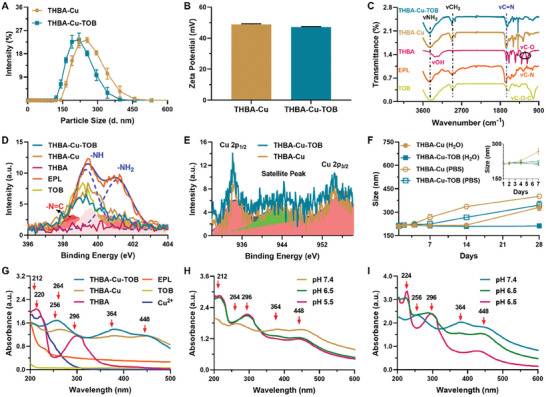
Characterization of THBA‐Cu and THBA‐Cu‐TOB NPs. A) Size distribution and B) zeta potential of THBA‐Cu‐TOB and THBA‐Cu‐TOB NPs. C) FTIR spectra of THBA, EPL, TOB, THBA‐Cu, and THBA‐Cu‐TOB NPs. D) High‐resolution N 1s XPS spectra of THBA, EPL, TOB, THBA‐Cu, and THBA‐Cu‐TOB NPs. E) High‐resolution Cu 2p XPS spectra of NPs. F) Size stability of NPs at various storage conditions. G) UV‐‐vis absorption spectra of THBA, EPL, TOB, Cu^2+^, THBA‐Cu, and THBA‐Cu‐TOB NPs. UV–vis absorption spectra of H) THBA‐Cu and I) THBA‐Cu‐TOB NPs at different pH values.

Next, a series of analytical techniques were used to investigate the formation mechanism, morphology, structure and stability of nanomaterials. As shown in **Figure** **2**A,B, the hydrodynamic size, polydispersity index (PDI), and zeta potential of NPs were explored through dynamic light scattering (DLS) measurements. The results indicated that the size and surface charge of THBA‐Cu‐TOB NPs at 220 ± 35 nm (PDI = 0.177) and +47.2 mV were very close to those of the drug‐free THBA‐Cu NPs (255 ± 40 nm, PDI = 0.215, +48.9 mV). Simultaneously, TEM and scanning electron microscope (SEM) images showed that the size of THBA‐Cu‑TOB NPs at the dried state was 182 ± 30 nm (Figure S2, Supporting Information). As for the Fourier transform infrared (FTIR) spectra measurement, the characteristic peak at 1632–1668 cm^−1^ represented the vibration absorption of C = N, which was attributed to the Schiff base reactions of the phenolic hydroxyl and aldehyde groups with amino groups.^[^ ^26^ ^]^ The peak at 1255 cm^−1^ of C–N bond in EPL shifted to 1263 cm^−1^ in THBA‐Cu and THBA‐Cu‐TOB NPs.^[^ ^27^ ^]^ Compared with THBA‐Cu, a wide peak at 942‐1088 cm^−1^ attributed to the C–O–C bond of TOB indicated the successful combination of TOB into THBA‐Cu‐TOB NPs (Figure 2C).^[^ ^28^ ^]^ Additionally, the peaks at 3300, 1300, and 1138 cm^−1^ appeared in THBA compounds, and disappeared after the coordination of phenolic hydroxyl groups with Cu^2+^ in the NPs.^[^ ^29^ ^]^

In addition, the X‐ray photoelectron spectroscopy (XPS) spectrum revealed the presence of C 1s, N 1s, O 1s, and Cu 2p peaks in THBA‐Cu‐TOB NPs (Figure S3A, Supporting Information), which was consistent with the elemental analysis in Figure 1B. As shown in Figure 2D, both high‐resolution N 1s peaks at 399.43 eV (–NH) and 401.23 eV (–NH_2_) of EPL obviously changed after the formation of NPs, which was attributed to the Schiff base reaction and coordination with Cu^2+^.^[^ ^30^ ^]^ A new peak at 398.53 eV (–N = C) of NPs could be attributed to the Schiff base reaction between aldehyde groups and amino groups.^[^ ^22^, ^31^ ^]^ Similarly, the C 1s peak at 285.85 eV (C–O and C–N) of TOB changed after the formation of THBA‐Cu‐TOB NPs (Figure S3B, Supporting Information), owing to the Schiff base reaction between amino groups in TOB and aldehyde groups.^[^ ^32^ ^]^ Meanwhile, three O 1s peaks in the THBA‐Cu‐TOB NPs were found at 531.53 eV (O = C), 532.23 eV (O–C), and 533.13 eV (HO–C), respectively (Figure S3C, Supporting Information),^[^ ^33^ ^]^ which demonstrated that the phenolic hydroxyl groups assigned to THBA was significantly weakened after the redox reaction, while the O–C peak in THBA‐Cu‐TOB NPs increased after loading the TOB. Furthermore, two dominated peaks at 934.13 and 954.53 eV were assigned to Cu 2p_3/2_ and Cu 2p_1/2_, respectively (Figure 2E).^[^ ^29^, ^34^ ^]^ Satellite peaks in the range of 937–948 eV were also observed, which were considered to be the typical peaks of Cu^2+^, verifying the coupled Cu^2+^ in the NPs.^[^ ^34^ ^]^ Specifically, the peaks at 546, 614, and 650 cm^−1^ and the peaks at 1444 and 1579 cm^−1^ observed in the spectrum of the THBA‐Cu and THBA‐Cu‐TOB NPs could be assigned to the coordination between Cu^2+^ and the oxygens of the galloyl in a high pH environment (Figure S4, Supporting Information).^[^ ^35^ ^]^

Subsequently, the dispersion stability of THBA‐Cu‐TOB NPs was evaluated by measuring their size changes in water and PBS solution for 28 days. As shown in Figure 2F, the size of THBA‐Cu NPs in an aqueous solution started to grow larger after 14 days of storage, while the size of THBA‐Cu‐TOB NPs kept intact after 28 days, indicating better dispersion stability of THBA‐Cu‐TOB NPs. In contrast, the size of THBA‐Cu NPs in the PBS solution significantly increased while THBA‐Cu‐TOB NPs only slightly increased after 7 days storage, mainly resulting from electrostatic adsorptions between the positively‐charged NPs and the negatively‐charged phosphate radicals. As shown in Figure 2G, the UV‐vis spectra showed no prominent peak for EPL and TOB, while Cu^2+^ had a weak peak at 220 nm. There were two prominent peaks at 212 and 296 nm in UV‐vis spectra of THBA, which was attributed to the polyphenol compounds. After the formation of NPs, the two peaks of THBA disappeared and new peaks appeared at 256, 264, 364, and 448 nm, suggesting the formation of quinones that participated in MPN, Schiff base reactions, and oxidative polymerization. Under neutral and alkaline conditions, polyphenols assembled with polymers and metals to develop NPs through coordination and oxidative crosslinking due to the strong and variable charge transfer bands.^[^ ^36^ ^]^

As for THBA‐Cu NPs, UV‐‐vis spectra showed that new peaks at 212 and 296 nm appeared at both pH 6.5 and 5.5, indicating the decomposition and release of THBA (Figure 2H). In contrast, the peaks at 256 and 364 nm in THBA‐Cu‐TOB NPs disappeared at pH 6.5, and the new peaks at 224 and 296 nm appeared, indicating the fracture of acid responsive chemical bonds and release of Cu^2+^ and THBA (Figure 2I). At pH 5.5, two prominent peaks at 224 and 296 nm appeared and the peaks at 256, 364, and 448 nm disappeared, demonstrating the disassembly of THBA‐Cu‐TOB NPs. Thus, in aqueous solution, THBA‐Cu‐TOB NPs significantly enhanced the storage stability of TOB due to the presence of a large number of amino acids in TOB. In weak acid (pH 6.5) to medium acid (pH 5.5) environments, nanomaterials showed significant pH sensitivity due to two types of acid responsive chemical bonds formation through MPN and Schiff base reactions.

### 2.2 Cu^2+^/TOB Release and Cell Compatibility

**Figure 3:**
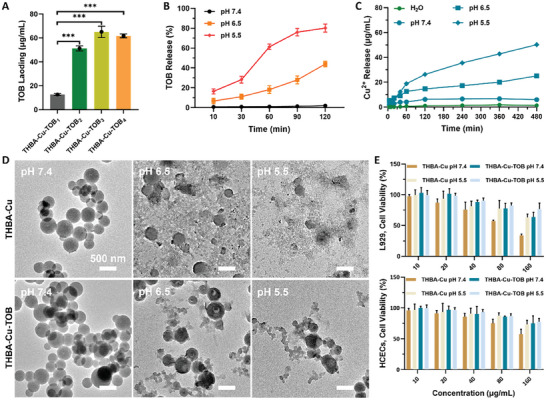
TOB delivery of THBA‐Cu‐TOB NPs and biocompatibility evaluation. A) TOB loading amount in THBA‐Cu‐TOB NPs under different TOB concentrations. B) TOB released from the THBA‐Cu‐TOB NPs under different pH values. C) Quantification of Cu^2+^ released from the THBA‐Cu‐TOB NPs in PBS at different pH values by ICP‐MS. D) TEM images of NPs after 2 h incubation at different pH values. E) Cell viability of L929 cells and HCECs at different pH values after incubation with different concentrations of NPs for 24 h.

The loading capacity of TOB into THBA‐Cu‐TOB NPs was studied. Different concentrations of TOB (0.5, 1.0, 1.5, and 2.0 mg mL^−1^) were added into the solution and reacted for 2 h before collection of the sediment by centrifugation. The content of TOB in synthesized THBA‐Cu‐TOB was 12.7, 51.2, 65.1, and 61.7 µg mL^−1^ named as THBA‐Cu‐TOB_1_, THBA‐Cu‐TOB_2_, THBA‐Cu‐TOB_3_, and THBA‐Cu‐TOB_4_, respectively, which were determined by spectrophotometry method after dialysis and aniline blue coloration (**Figure** **3**A; Figure S5, Supporting Information). TEM images showed that the THBA‐Cu‐TOB NPs with different TOB loading were spherical with uniform size (Figure S6, Supporting Information). However, when the feed concentration of TOB was 2.0 mg mL^−1^, the product undergone obvious precipitation in the solution, indicating poor stability of THBA‐Cu‐TOB_4_. Therefore, THBA‐Cu‐TOB_3_ was selected for subsequent experiments based on its high drug loading capacity and good dispersibility and, the release of TOB was also measured to explore the drug release behavior at different pH values (7.4, 6.5, and 5.5). It was found that THBA‐Cu‐TOB maintained very high stability and almost no drug was released at pH 7.4, which could be attributed to the good stability in such pH value (Figure 3B). However, the drug release significantly accelerated in acidic environments that released 11.1% and 28.2% at 30 min and 43.9% and 80.2% at 2 h at pH 6.5 and 5.5, respectively. The release process of Cu^2+^ from THBA‐Cu‐TOB NPs under different pH conditions through inductively coupled plasma‐mass spectrometry (ICP‐MS) testing certified significant pH sensitivity of the metal‐polyphenol bonding formed in nanomaterials (Figure 3C). In addition, pH sensitive Schiff base bonds in THBA‐Cu‐TOB NPs could effectively lead to the rapid TOB release responsive to acidic pH value.

Subsequently, TEM measurement was applied to track the morphology and size changes of nanomaterials in the degradation process. It was also found that the size of THBA‐Cu‐TOB NPs gradually decreased during TOB release. Further research on the release of Cu^2+^ revealed that the release of Cu^2+^ did not significantly accelerate under pH 6.5 conditions (Figure S7, Supporting Information). At pH 5.5, although the release of Cu^2+^ is significantly accelerated, it is still not fully released after 8 h. Therefore, it is reasonable to infer that the chemical bonds formed in Schiff base reaction between TOB and THBA were more sensitive to acidic pH value than the formed metal‐polyphenol chelating forces between Cu^2+^ and THBA. The stability of nano‐drug delivery system and its responsiveness to disease microenvironment is a contradiction need to be regulated. The low concentration release of antibiotics from drug carrier with low stability cannot effectively kill bacteria and also leads to bacterial resistance. On the other hand, if the drug carrier is too sensitive to the environment, it may be completely released before penetration into the interior of biofilm to achieve effective removal and bacterial killing. Therefore, an ideal antibiotic nano‐drug delivery system needs to be stable in a physiological environment (pH 7.4), and gradually explodes during the penetration into biofilm to kill the bacteria throughout the entire biofilm. As shown in Figure 3D, TEM images showed that the THBA‐Cu NPs entirely degraded at pH 5.5 after 2 h incubation. In contrast, THBA‐Cu‐TOB NPs still maintained a certain nano morphology with a delayed degradation process after 2 h incubation. Therefore, the loading of TOB further significantly improved the stability of the as‐prepared NPs.

Furthermore, the cytotoxicity of THBA‐Cu‐TOB NPs toward L929 and HCECs was evaluated before and after TOB release (Figure 3E). It was found that the THBA‐Cu‐TOB NPs showed high cell viability to L929 (77.9%) and HCECs (85.9%) even the concentration was up to 80 µg mL^−1^. In comparison, the release of TOB showed negligible cytotoxicity, and the cell activities (82.6% and 87.0% to L929 and HCECs, respectively) were even higher than that before TOB release at the 80 µg mL^−1^ concentration, which might be due to the high positive charge properties (+48.9 mV and +47.2 mV for THBA‐Cu and THBA‐Cu‐TOB, respectively) of nanomaterials (Figure 2B) and the protective effect of released polyphenols on cell viability. Therefore, the achievement of cellular activity confirmed the low toxic effect on mammalian cells during the delivery process and after disassembly. The hemolysis test also was applied to examine the blood compatibility of THBA‐Cu‐TOB NPs (80 µg mL^−1^) with THBA‐Cu NPs as the control. The result certified the excellent blood compatibility of THBA‐Cu‐TOB NPs with hemolysis ratios were 3.6% and 4.6% before and after TOB release, respectively (Figure S8, Supporting Information).

### 2.3 Antibacterial Activity of THBA‐Cu‐TOB NPs

First, the checkerboard method was used to evaluate the synergetic effect of THBA, Cu^2+^, and EPL on TOB in killing *P. aeruginosa* (Figure S9, Supporting Information). It was found that both THBA and Cu^2+^ showed no bactericidal effect even the concentration was up to 256 µg mL^−1^, as well as combined with TOB. Nevertheless, EPL containing amino groups exhibited significant bactericidal effect when the concentration reached 32 µg mL^−1^ and could eliminate bacteria at the concentration of 128 µg mL^−1^, leading the reduction of OD from 1.0 to 0, which could be owing to the cationic feature of EPL in damage of bacterial membrane.^[^ ^37^ ^]^ At the same time, EPL showed an obvious synergistic bactericidal effect in conjunction with TOB. During the growth of bacteria, organic acids as the metabolite led to the acidification of the environment. The initial pH of the bacterial culture medium was 7.15, and decreased to 6.8 after 24 h cultivation of *P. aeruginosa* (Figure S10, Supporting Information). Importantly, the internal pH of the biofilm is much lower even less than 5.5.^[^ ^38^ ^]^ Therefore, the MIC and MBC were tested at pH 6.8 and 5.5 respectively, to evaluate the antibacterial performance of THBA‐Cu‐TOB NPs. As shown in Table S3 (Supporting Information), the THBA‐Cu NPs showed negligible bactericidal ability with a high MIC (> 250 µg mL^−1^) and MBC (> 250 µg mL^−1^). In contrast, the THBA‐Cu‐TOB NPs had excellent bacteriostatic and bactericidal effects with MIC at 8 and 4 µg mL^−1^ and MBC at 8 and 8 µg mL^−1^ at pH 6.8 and 5.5, respectively.

**Figure 4:**
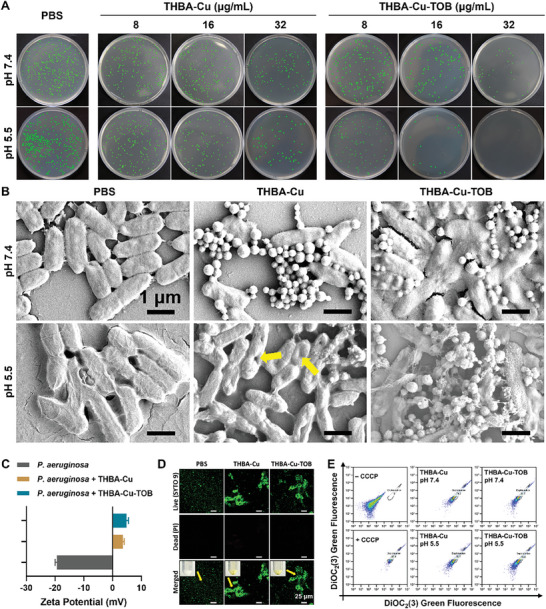
In vitro antibacterial activity against *P. aeruginosa*. A) Representative photos of *P. aeruginosa* colonies on LB agar broth plates after being treated with PBS (control) and various concentrations of NPs at different pH values. Green represents *P. aeruginosa* colonies. B) SEM images of *P. aeruginosa* cells after being treated with PBS and NPs (40 µg/mL) at different pH values for 2 h, respectively. C) Zeta potential of *P. aeruginosa* (10^6^ CFU/mL), and *P. aeruginosa* treated by THBA‐Cu and THBA‐Cu‐TOB, respectively. D) CLSM images of the *P. aeruginosa* incubated with PBS, THBA‐Cu and, THBA‐Cu‐TOB (yellow arrows showed bacterial aggregation). Live bacteria: green (SYTO‐9); Dead bacteria: red (propidium iodide, PI). E) Flow cytometry analysis of the membrane potential of *P. aeruginosa* cells after 2 h staining by DiOC_2_(3).

The bactericidal activity of THBA‐Cu‐TOB NPs at different concentrations was further evaluated by plate colony count method against *P. aeruginosa* (**Figure** **4**A; Figure S11, Supporting Information). The PBS treatment group was taken as the control with the colony‐forming units (CFU) being constantly kept at 6.51 lg CFU mL^−1^ (pH 7.4) and 6.50 lg CFU mL^−1^ (pH 5.5) respectively, indicating the good survival status of bacteria in physiological environment. The THBA‐Cu NPs showed low bactericidal performance even when the concentration was up to 32 µg mL^−1^ with bacteria concentrations at 6.08 lg CFU mL^−1^ and 5.76 lg CFU mL^−1^ at pH 7.4 and 5.5, respectively (the bactericidal ratio at 6.6% and 11.5%, respectively). In comparison, the THBA‐Cu‐TOB NPs showed obvious pH‐ and concentration‐dependent bactericidal activity. Even when the concentration was 32 µg mL^−1^ at pH 7.4, bacteria concentration and bactericidal ratio were 5.45 lg CFU mL^−1^ and 16.3%, respectively, which could be attributed to the high stability of nanomaterials under pH 7.4 condition with little release of TOB. However, at pH 5.5, the bactericidal activity significantly increased at concentrations of 16 µg mL^−1^ and 32 µg mL^−1^ with bactericidal ratios at 94.7% and 99.9%, respectively. Notably, the decrease of bacteria number was greater than 6 orders of magnitude when the concentration of THBA‐Cu‐TOB NPs was 32 µg mL^−1^. The stimuli‐responsive rapid release of TOB and the synergistic effect of EPL were the basic reasons leading to bacterial death.

The adsorption of cationic NPs onto *P. aeruginosa* and morphological changes of bacteria treated in different pH environments were observed through SEM images (Figure 4B). It was found that the bacteria remained normal morphology in PBS buffers at pH 7.4 and 5.5. At pH 7.4, the cationic THBA‐Cu NPs and THBA‐Cu‐TOB NPs adsorbed on the negatively charged bacterial membrane surface through electrostatic interactions. THBA‐Cu NPs were unstable at pH 5.5, and rapidly degraded into THBA, Cu^2+^, and EPL owing to the fracture of metal‐polyphenol and Schiff base bonds. The rapid release of EPL also caused some bacterial structures to be dented and damaged as indicated by the yellow arrow. In contrast, there were still THBA‐Cu‐TOB NPs with reduced size adhering to the surface of the bacteria after 2 h of incubation. At pH 5.5, EPL and TOB were released by acidic responsive degradation of THBA‐Cu‐TOB NPs, which acted synergistic bactericidal effect on *P. aeruginosa*. Notably, the participation of TOB in THBA‐Cu‐TOB NPs assembly increased the bonding forces between the components, which achieved an appropriate balance between stability and responsiveness in acidic environments. An unstable drug delivery system not only leads to drug loss but also easily causes bacterial drug resistance. A drug delivery system without responsiveness cannot achieve high concentrations of drugs at the lesion site to eliminate the bacteria, especially for bacteria inside biofilm, which was due to the poor penetration of the free drugs and the difficulty in eradicating drug‐resistant bacteria.

As shown in Figure 4C, zeta potential of initial *P. aeruginosa* was −19.4 mV. After incubation with THBA‐Cu NPs and THBA‐Cu‐TOB NPs, the zeta potential of *P. aeruginosa* changed to +3.6 m and +4.9 mV, respectively. Confocal laser scanning microscope (CLSM) images showed that the bacteria in PBS group were uniformly dispersed (Figure 4D). On the contrary, bacteria aggregation occurred in THBA‐Cu NPs and THBA‐Cu‐TOB NPs treatment groups, indicating the strong electrostatic interactions between cationic NPs and negatively charged bacterial membrane. As shown in Figure 4E, *P. aeruginosa* without carbonyl cyanide 3‐chlorophenylhydrazone (CCCP) had undamaged membrane and exhibited relatively strong red fluorescence signals. A triangle flow cytometry gate was drawn to mark the bacteria with completely depolarized cell membrane in +CCCP group. Upon incubated with cationic THBA‐Cu and THBA‐Cu‐TOB, the proportion of green fluorescence increased over 74.0% in pH 5.5, indicating that the integrity of the membrane was impaired. Therefore, cationic NPs can increase the permeability of bacteria cell membrane, thereby enhancing the curative effect of antibiotic synergistic therapy. Furthermore, the aggregation between bacteria and nanocarriers is more conducive to the high concentration release of local drugs and the killing of bacteria. It is worth mentioning that this killing method is very similar to the action mode of neutrophils producing extracellular trap (NET) network to kill pathogenic bacteria through the suicidal NETosis process.^[^ ^39^ ^]^

**Figure 5:**
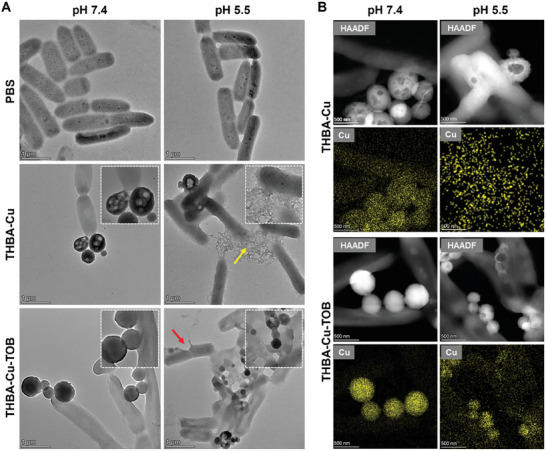
The electrostatic interactions of NPs to *P. aeruginosa*. A) TEM images of *P. aeruginosa* after 2 h incubation with NPs at different pH values. Magnified images showed the adherence of NPs on bacterial surface. B) HAADF‐STEM images of *P. aeruginosa* after 2 h incubation with NPs at different pH values.

Furthermore, the interaction between nanomaterials and bacteria and bactericidal mechanism were further studied using TEM. As shown in **Figure** **5**A, TEM images showed that the morphology of *P. aeruginosa* in PBS was normal at pH 7.4 and 5.5. At pH 7.4, both THBA‐Cu NPs and THBA‐Cu‐TOB NPs were adsorbed onto *P. aeruginosa* surface and mainly maintained their sizes. At pH 5.5, most THBA‐Cu NPs degraded owing to the fracture of pH responsive metal‐polyphenols and Schiff base bonds. In contrast, the size of THBA‐Cu‐TOB NPs greatly reduced into smaller irregular products like fragments after bomb explosion, and it was clearly seen that the morphology of some bacteria was highly damaged, which were simultaneously affected by the release of TOB and EPL. Hollow *P. aeruginosa* was also observed caused by the outflow of the content and part of the membrane of bacteria was even torn (indicated by red arrows). As shown in Figure 5B, the higher concentration of Cu^2+^ in THBA‐Cu‐TOB NPs, the denser the sphere formed, while the lower the concentration of Cu^2+^ in THBA‐Cu NPs, the looser the structure of THBA‐Cu NPs. The distribution of Cu element also revealed the degradation process of nanomaterials and the interactions between material components and bacteria during degradation. In the pH 5.5 environment, almost all THBA‐Cu NPs degraded and lost their original structures. While the size of THBA‐Cu‐TOB NPs significantly decreased, but some nanomaterials still maintained sphericity. Therefore, the doping of TOB further enhanced the bonding ability between components, improved stability under acidic conditions, and provided time for drug delivery to the entire lesion site and the biofilm. In addition, the surface of bacteria was covered with Cu^2+^, indicating that the targeting effect of bacterial based on electrostatic interactions significantly increased the concentration of drug acting on bacteria.

### 2.4 Biofilm Penetration and Bacterial Sterilization

**Figure 6:**
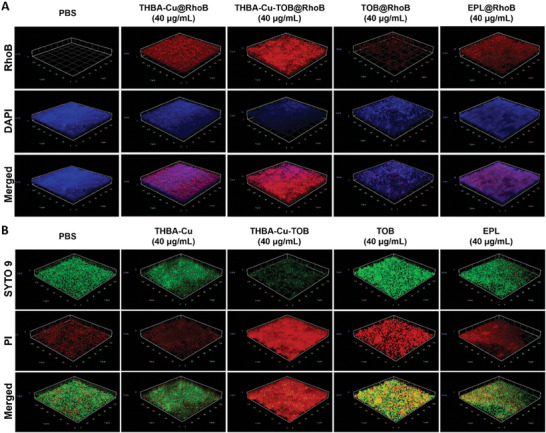
*P. aeruginosa* biofilm penetration and eradication by THBA‐Cu‐TOB NPs. A) Biofilm permeability of fluorescently labeled NPs and drugs. DAPI: blue; RhoB: red. B) Live & dead bacterial staining of the biofilm treated by NPs and drugs. Live bacteria: SYTO‐9, green; Dead bacteria: PI, red.

Rhodamine B (RhoB) labeled NPs and agents including THBA‐Cu@RhoB, THBA‐Cu‐TOB@RhoB, TOB@RhoB, and EPL@RhoB were incubated with *P. aeruginosa* biofilm for 2 h, respectively. The incubated THBA‐Cu‐TOB@RhoB NPs and THBA‐Cu‐TOB NPs showed similar morphology, size, and zeta potential (Figure S12, Supporting Information). As shown in **Figure** **6**A; Figures S13 and S14 (Supporting Information), TOB@RhoB showed extremely low penetration into the *P. aeruginosa* biofilm, and only a small amount of TOB distributed a few microns from the surface of the biofilm, which indicated that free antibiotic drugs were difficult to remove the biofilm.^[^ ^40^ ^]^ Meanwhile, the free EPL also exhibited low penetration owing to the strong retention effect of viscous liquid biofilm on it. Interestingly, THBA‐Cu NPs could efficiently penetrated into the biofilm, which might be caused by their good hydrophilicity and small size to reduce the retention effect by bacterial biofilm mucus systems. However, due to the high sensitivity to pH value, THBA‐Cu NPs only reached half depth of the biofilm (≈10 µm). Compared with THBA‐Cu NPs, THBA‐Cu‐TOB NPs showed excellent biofilm permeability due to the positive charge, structural stability, and suitable nano‐size.^[^ ^24^ ^]^

Then, Live/Dead bacterial staining method was used to evaluate the biofilm eradication ability of THBA‐Cu‐TOB NPs (Figure 6B). It was found that most of the bacteria treated by PBS were stained with green fluorescence, indicating the living status of the bacteria. Only a small number of dead bacteria stained with red fluorescence were the normal metabolic death of some bacteria in the biofilm. Both THBA‐Cu and EPL showed low bactericidal ability against *P. aeruginosa* within the biofilm, which could be attributed to the low permeability and lack of bactericidal components. For the free TOB treatment group, even when the drug concentration reached 40 µg mL^−1^, most of the bacteria in the biofilm were still in a viable state. Therefore, the low permeability of drug into biofilm is a key factor limiting the bactericidal effect. Only when the TOB concentration reached 160 µg mL^−1^ (Figure S15, Supporting Information), the bactericidal effect was similar to that of THBA‐Cu‐TOB NPs treatment group (40 µg mL^−1^). However, the cytotoxicity caused by high concentrations of drug could not be ignored at such concentration. Notably, the equivalent TOB concentration in THBA‐Cu‐TOB NPs treated group was only 2.6 µg mL^−1^, which was only 1.6% of the free TOB (160 µg mL^−1^). The excellent bactericidal performance of nano drug delivery system to the bacteria within the biofilm should be attributed to the delicate balance of stability and responsiveness under acidic condition.

### 2.5 The Antioxidant, Anti‐Inflammation, and Cell Migration Tests

**Figure 7:**
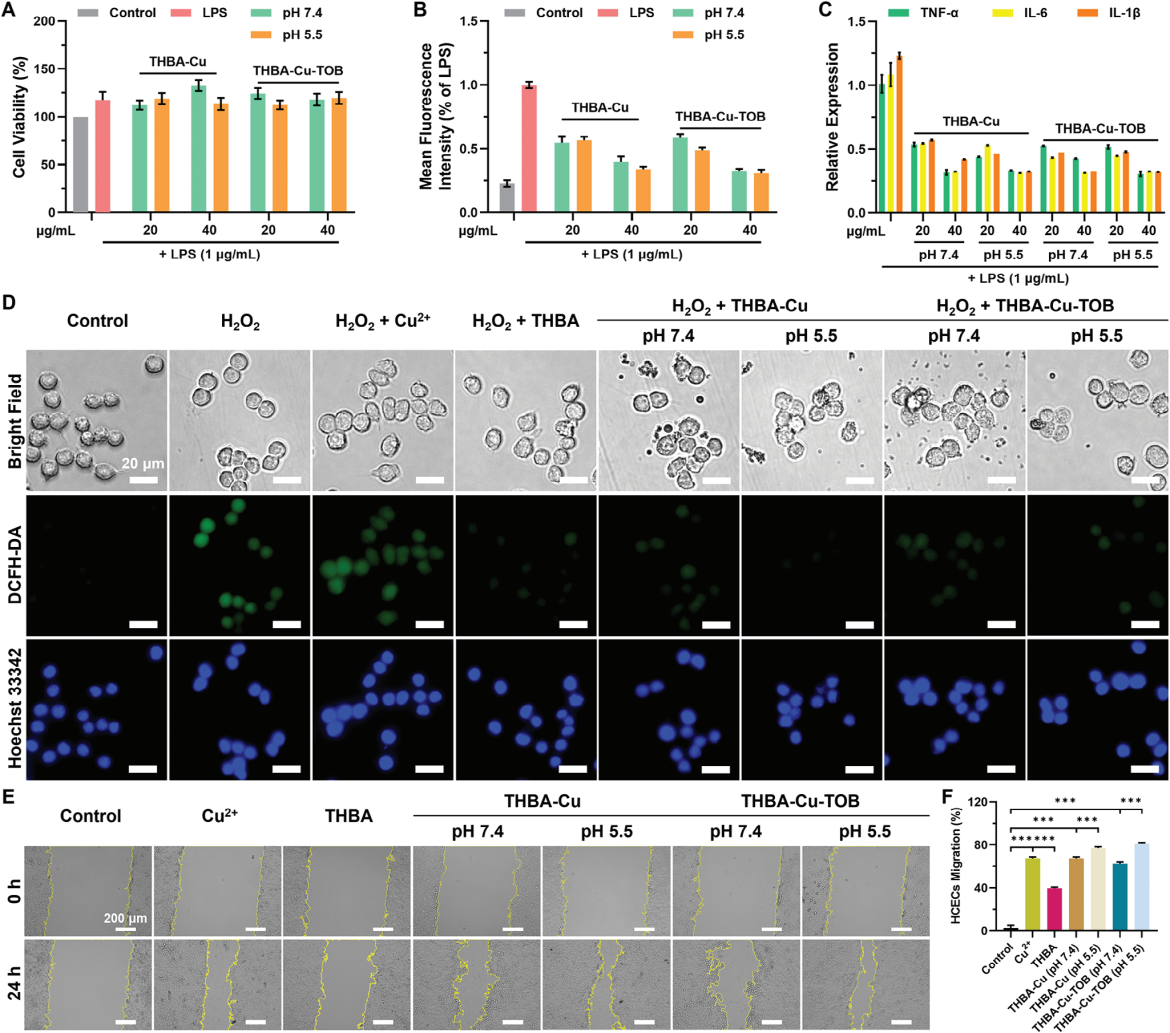
In vitro antioxidant activity and cell migration of HCECs treated by THBA‐Cu‐TOB NPs. A) Cell viability of RAW264.7 cells simultaneously treated by LPS and NPs. B) Detection and suppression of LPS‐induced ROS production in microphage cells by flow cytometry using DCFH‐DA fluorescent probe. Data were presented as means ± SD (*n* = 5). C) Anti‐inflammatory activity of NPs on LPS‐induced RAW264.7 cells at different pH values. D) ROS production in RAW264.7 cells induced by H_2_O_2_ (50 µm) after incubation with NPs. The cells were labeled with DCFH‐DA (green) and cell nuclei was labeled with Hoechst 33 342 (blue). E) Typical images of HCECs incubation with NPs for 24 h. F) Quantification of HCECs migration. Data were presented as means ± SD (*n* = 3): ***p < 0.001.

Macrophages generated inflammatory factors when activated by endotoxin LPS.^[^ ^41^ ^]^ First, the biocompatibility of nanomaterials on macrophages was explored through the CCK‐8 method before and after nanomaterials degradation through incubation with RAW264.7 cells for 24 h. Compared with PBS treated group, the cell viability of macrophages in LPS and NPs treated groups was even higher, showing negligible toxic effect to macrophages (**Figure** **7**A). Under normal circumstances, cells maintain a dynamic stability between the production and clearance of ROS with the assistance of enzymes and antioxidant substances. At low concentrations, ROS plays an important physiological function as a signaling molecule in regulating cell proliferation and growth. However, when infection and inflammation occur, oxidative stress is one of the indispensable self‐protection mechanisms of the body's immune system. In addition, due to various reasons causing damage to the antioxidant defense system, the intracellular levels of ROS in the tissue will also increase, leading to oxidative stress. However, at high concentrations, ROS can modify or even damage essential macromolecules, such as proteins, ribonucleic acids, and lipids. Therefore, maintaining an appropriate level of ROS to achieve redox homeostasis is crucial to cell normal function and body health. LPS is a common endotoxin secreted by bacteria, which causes oxidative stress reactions in macrophages. In this nanoplatform, the release of THBA after degradation of nanomaterials achieved the removal of ROS based on the reducibility of polyphenol groups using DCFH‐DA as fluorescent probe (Figure 7B). Furthermore, in the physiological environment and the infected acidic microenvironment, THBA‐Cu‐TOB NPs and THBA‐Cu NPs represented similar ROS clearance capacity.

Oxidative stress and inflammatory response are two weapons of the immune system that coexist and promote each other.^[^ ^42^ ^]^ When the oxidative stress state of macrophages is eliminated, the inflammatory response will also undergo corresponding changes. Quantitative analysis of inflammatory factors expression was performed to explore the inflammatory response status of macrophages and real‐time quantitative polymerase chain reaction (qPCR) was used to detect the mRNA expression of inflammatory factors. As shown in Figure 7C, a high level of mRNA expressions of inflammation factors including TNF‐α, IL‐6, and IL‐1β were shown in the control group without any treatment. In contrast, both nanomaterials exhibited a significant ability to reduce inflammatory response in a concentration‐dependent manner. It was also found that the pH value and TOB loading had little effect on the anti‐inflammatory function. When the concentration of THBA‐Cu‐TOB NPs was 40 µg mL^−1^ (pH 7.4), the expressions of TNF‐α, IL‐6, and IL‐1β significantly decreased.

In vitro macrophages oxidative stress model induced by hydrogen peroxide (H_2_O_2_) was used to further evaluate the antioxidant capacity of NPs. As shown in Figure 7D, the macrophages in both untreated and Cu^2+^ treated groups glowed strong green fluorescence, indicating the production of endogenous ROS. In contrast, no green fluorescence was observed in the THBA (40 µg mL^−1^) treated group, indicating that free THBA could significantly inhibit the ROS production in macrophages. Furthermore, the cells in the THBA‐Cu‐TOB NPs and THBA‐Cu NPs treated groups emitted very weak green fluorescence, indicating the excellent ROS inhibition effect of THBA. The effect of NPs on HCECs migration was detected by scratch test. As shown in Figure 7E,F, the migration rate of HCECs in the control group was 5.3% after 24 h incubation. However, the migration rates of HCECs in the Cu^2+^ and THBA treated groups were 68.8% and 38.5%, respectively, which was mainly caused by the promotion of Cu^2+^ and THBA.^[^ ^43^ ^]^ Cu^2+^ can be used by copper dependent enzymes or proteins, which play a signaling function to promote HCECs migration.^[^ ^33^, ^44^ ^]^ Meanwhile, the role of THBA in promoting cell migration was owing to its regulatory role in the balance of oxidative stress of cells around the “scratch wound”. In addition, the nanomaterials preferably promoted the migration of HCECs with the migration rates at 66.3% and 60.9% before degradation, 78.3% and 79.8% after degradation for THBA‐Cu NPs and THBA‐Cu‐TOB NPs, respectively. Hence, THBA‐Cu‐TOB NPs effectively scavenged ROS, reduced inflammation and promoted HCECs migration, which could play a critical role in corneal ulcer healing.

### 2.6 In Vivo Evaluation through Infectious Corneal Ulcer Model

Before taking the in vivo evaluation through infectious corneal ulcer model, the biosafety of the THBA‐Cu‐TOB NPs was evaluated by pathological section, blood routine, liver, and kidney function indices methods. The hematoxylin and eosin (H&E) staining images showed that the cornea, heart, liver, spleen, lung, and kidney cells kept intact morphology, and no inflammatory cells were observed (e.g., necrotic cells and macrophages) (Figure S16A, Supporting Information). Meanwhile, there was also no noticeable pathological change in the major organs. Compared with the control group, no significant differences in liver and kidney biochemical indexes, white blood cells, red blood cells, platelets, and other blood indexes in the THBA‐Cu‐TOB NPs treated group (Figure S16B, Supporting Information), indicating the non‐toxic in hematology of THBA‐Cu‐TOB NPs. Therefore, the THBA‐Cu‐TOB NPs displayed safety in treating infectious corneal ulcer as ophthalmic eye drops.

**Figure 8:**
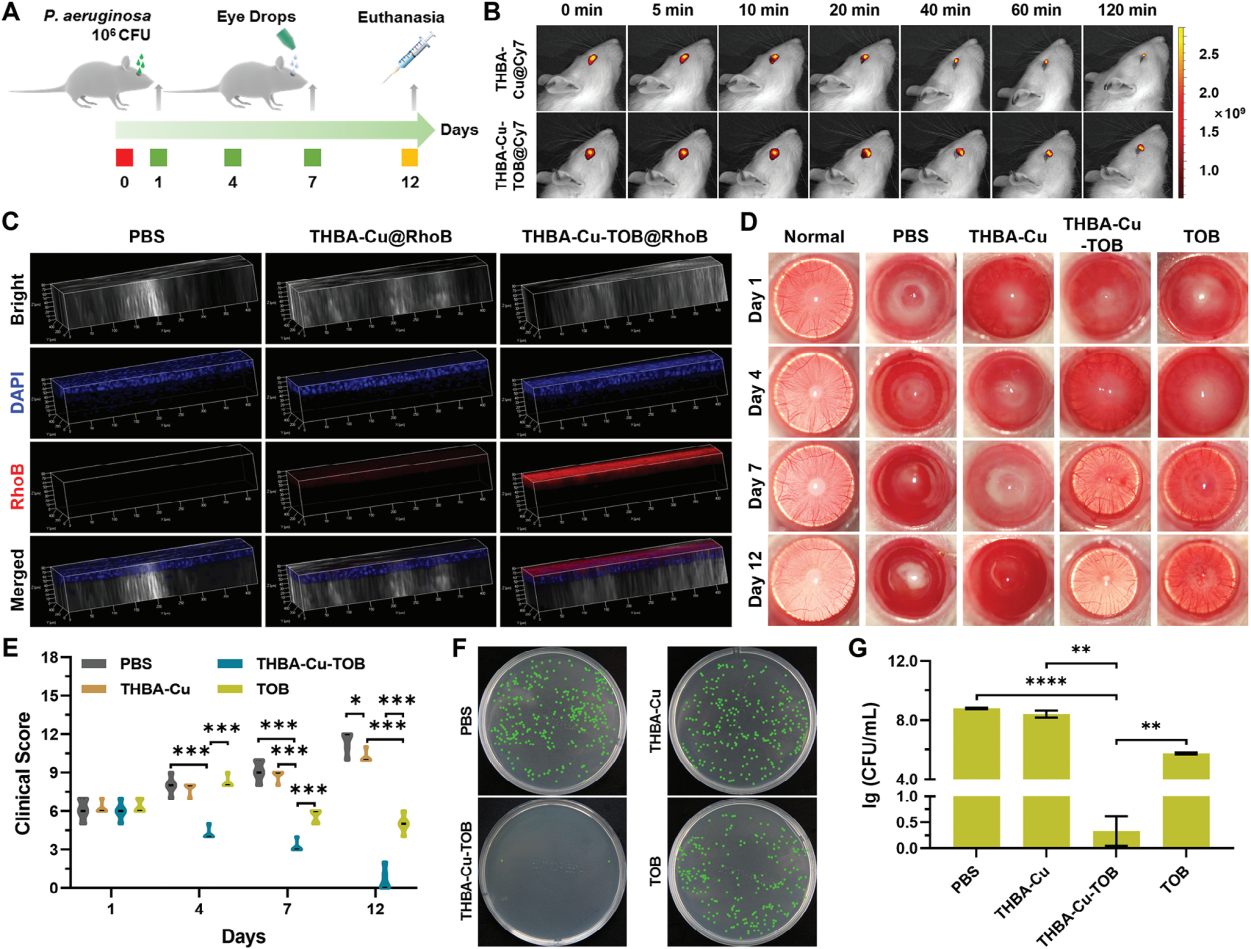
In vivo therapeutic efficacy of NPs. A) Schematic illustration of establishment of *P. aeruginosa*‐induced corneal wound model in rats and their treatment with eye drops. B) IVIS optical images of Cy7‐labeled NPs eye drops in eyes at different time points. C) CLSM images of RhoB‐labeled NPs in corneal tissues. D) Slit‐lamp observation of the clinical signs (Iris hyperemia, miosis, exudation, hypopyon, *etc*.) of eyes and E) the clinical scores of infection severity from each group. F) Typical photos of *P. aeruginosa* colonies on agar broth plates after being treated with PBS, NPs, and TOB, respectively. Green represents *P. aeruginosa* colonies. G) Determination of the number of viable bacteria per milliliter of bacteria by colony counts in different groups. Data were presented as means ± SD (*n* = 3): *p < 0.05, **p < 0.01, ***p < 0.001, and ****p < 0.0001.

After *P. aeruginosa* infected and infiltrated in the corneal wound, the wound rapidly developed into infectious corneal ulcer owing to the high penetration pressure of *P. aeruginosa*.^[^ ^45^ ^]^ If left untreated, it will lead to persistent corneal infections, bacterial biofilm formation, chronic inflammatory reactions, and corneal tissue damage. After successful establishment of corneal ulcer model, different treatment methods were used to treat the corneal ulcer until euthanasia 12 days later (**Figure** **8**A). First, the retention of NPs on ocular surface was detected by in vivo optical imaging system. As shown in Figure 8B, the fluorescence intensity declined to 30% and 65% for THBA‐Cu and THBA‐Cu‐TOB NPs treated groups after 2 h, respectively, compared to the initial fluorescent intensity (Figure S17, Supporting Information), indicating the higher stability and retention of THBA‐Cu‐TOB NPs. Subsequently, the penetration ability of NPs was further observed by CLSM (Figure 8C). The electrostatic interactions between positively charged nanomaterials and negatively charged corneal surface and nano size effect improved the corneal adhesion and permeability of NPs.^[^ ^7^, ^46^ ^]^ Furthermore, THBA‐Cu‐TOB NPs displayed a much higher corneal permeability than that of THBA‐Cu NPs due to the enhanced stability of TOB couplings with THBA through chemical bonds.

The slit lamp and clinical score were used to evaluate the changes of inflammatory response and transparency after treatment (Figure 8D,E). The results illustrated that the normal cornea maintained good transparency under the experimental growth conditions without infections and inflammatory reaction. As for the bacteria infected model established groups, the corneas showed obvious corneal infection and ulceration in PBS, THBA‐Cu NPs, THBA‐Cu‐TOB NPs, and TOB treated groups with consistent clinical score at 6 points, which seriously affected the transparency of the cornea at day 1. At day 4 (three days after the treatment), obvious necrotic zone and a large amount of purulent secretion appeared in the corneas in PBS, THBA‐Cu‐TOB NPs, and TOB treated groups with a similar clinical score higher than 8 points, indicating the aggravated inflammatory response. In contrast, only in the THBA‐Cu‐TOB NPs treated group, the corneal ulcer state was significantly reduced, leading to the increase of transparency and reduction of clinical score below 5 points. At day 7, as the development of ulcers and dropping of necrotic tissues, perforation and iris prolapse fragments appeared in the center of necrotic corneal tissue in the PBS and THBA‐Cu NPs treated groups with the clinical scores increased to 9 points. As for the TOB treated group, despite significant improvement in corneal ulcer was observed, the cornea was still in a severe inflammatory reaction and edema state. The inflammation score showed that it still maintained similar clinical score to that just establishing the animal model (≈6 points) even after 11 days treatment. At day 12, serious ulcer developed in PBS and THBA‐Cu NPs treated groups, and the whole corneal tissue turned into necrotic with the clinical score higher than 10 points. In contrast, the corneas in the THBA‐Cu‐TOB NPs treated group basically returned to normal corneal state with a high transparency and disappearance of ulcer lesions. As shown in Figure S18 (Supporting Information), the weight of rats in the THBA‐Cu‐TOB group remained normal owing to the effective infection treatment.

Furthermore, the corneal tissue in each group was collected for plate counting of the bacterial concentration at day 12. As shown in Figure 8F,G, the plate counting results indicated that a large number of bacteria were found in PBS, THBA‐Cu NPs, and TOB treated groups, indicating weak bactericidal effect in corneal tissue. In comparison, almost no bacteria were found in the THBA‐Cu‐TOB NPs treated group. Further quantitative statistical showed that the bacteria concentration in PBS and THBA‐Cu treated groups were 8.79 lg CFU mL^−1^ and 8.40 lg CFU mL^−1^, respectively. The number of bacteria in the TOB treated group was significantly reduced compared to that in the PBS treated group (a decrease of 3.04 lg CFU mL^−1^). However, the cornea still showed a large number of defects after 11 days of treatment, indicating delayed inflammatory damage during treatment. The number of bacteria in the THBA‐Cu‐TOB group was only 0.33 lg CFU mL^−1^, which revealed 8 orders of magnitude reduction in bacteria number. These results indicated that the THBA‐Cu‐TOB NPs possessed an excellent in vivo bactericidal effect against *P. aeruginosa*. Therefore, compared to free TOB, the delivery of nanomedicines significantly improved the bioavailability of TOB. The significantly reduced inflammatory response by THBA‐Cu‐TOB NPs was attributed to the excellent retention on cornea, bacterial biofilm permeation, and bactericidal capabilities. As the infection was promptly eliminated, the released THBA components played a role in ROS clearance and anti‐inflammatory, comprehensively exerting the therapeutic effect on corneal ulcer healing.

### 2.7 Histopathological and Immunofluorescence Evaluation

**Figure 9:**
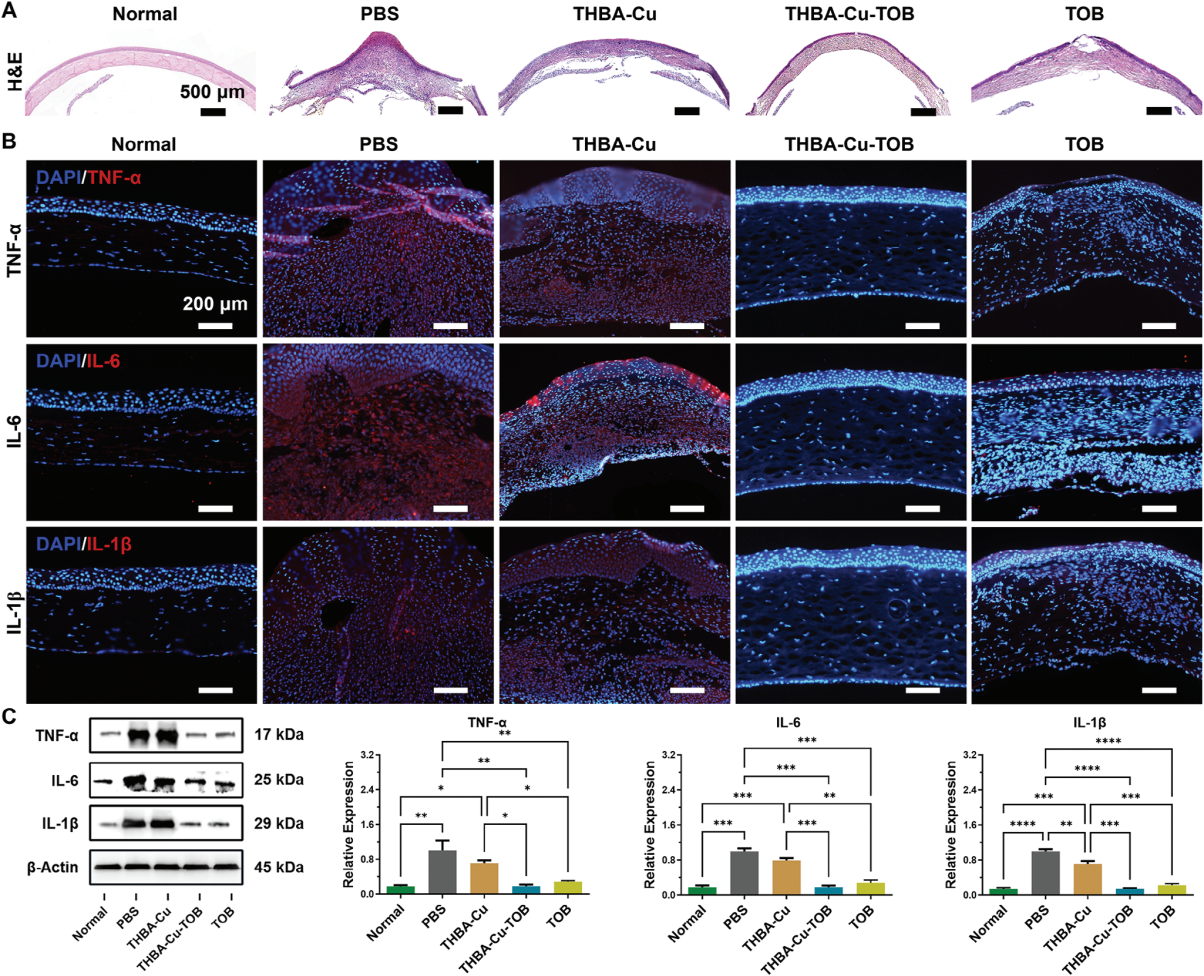
Inflammatory marker expressions of the corneal wound after different treatments. A) H&E staining images of infected corneal tissues in each group. B) Immunofluorescent staining for TNF‐α, IL‐6, and IL‐1β of infected corneal tissues in each group (Blue: DAPI; Red: inflammatory cytokines). C) Western blotting analysis of TNF‐α, IL‐6, IL‐1β, and β‐actin protein expressions in corneal tissues in each group. Data were presented as means ± SD (*n* = 3): *p < 0.05, **p < 0.01, ***p < 0.001, and ****p < 0.0001.

Corneal tissue H&E and immunofluorescence staining were used to detect the morphological structure, inflammatory cell infiltration, and expression of inflammatory factors after different treatments. As shown in **Figure** **9**A, H&E staining results showed that a large number of inflammatory cells infiltration and tissue structure destruction in the PBS and THBA‐Cu NPs treated groups. Although inflammatory cells infiltration was significantly alleviated in the free TOB eye drop treatment group, the central corneal tissue was significantly damaged. The overall corneal thickness was uneven in these three control groups and even huge corneal protrusion and vacuole were found in PBS and TOB treated groups. Only in the THBA‐Cu‐TOB NPs treated group, the corneal structure was intact with mild inflammatory cells infiltration and no obvious corneal epithelial defect. Furthermore, the immunofluorescence staining of inflammatory cytokines including TNF‐α, IL‐6, and IL‐1β were used to evaluate the inflammatory levels after different treatments (Figure 9B). Almost no red immunofluorescence was observed in the THBA‐Cu‐TOB NPs treated group, indicating disappearance of keratitis reaction and intact structural morphology similar to normal cornea. Although no obvious inflammatory response was found in the free TOB treated group, there were numerous defects in the corneal epithelial and endothelial cell layers, indicating the significantly affected refractive function of the cornea. Besides, the cornea was still in intense inflammatory response state with a large number of pro‐inflammatory cytokines including TNF‐α, IL‐6, and IL‐1β almost covered the entire cornea, which could lead to corneal edema, inflammatory cells infiltration, prolonged corneal ulcers healing, and tissue structural damage.

As shown in Figure 9C, western blotting results showed the protein expressions of the pro‐inflammatory factors in PBS and THBA‐Cu NPs treated groups were significantly higher due to the lack of bactericidal ingredients. In comparison, the protein expressions of the pro‐inflammatory factors in THBA‐Cu‐TOB NPs and TOB treated groups almost returned to the initial level of normal cornea. Due to the strong invasiveness and biofilm formation of *P. aeruginosa*, the bacteria cannot be completely destroyed by the body's own immune function alone (PBS treatment group). Within 11 days of self‐healing, there was still a large amount of bacteria and intense levels of inflammatory response. Similarly, the release of THBA and Cu^2+^ in THBA‐Cu NPs group to the ulcer area also showed no obvious effect in corneal ulcer healing. The THBA‐Cu‐TOB NPs showed both stability and responsiveness to infected microenvironment for prolonged ocular surface retention and corneal barrier penetration based on the positive charge property and nano size effect. Consequently, THBA‐Cu‐TOB NPs achieved the repair of refractory corneal ulcer through synergistic sterilization and anti‐inflammatory effects.

### 2.8 Anti‐Inflammatory Signaling Pathway Investigation

**Figure 10:**
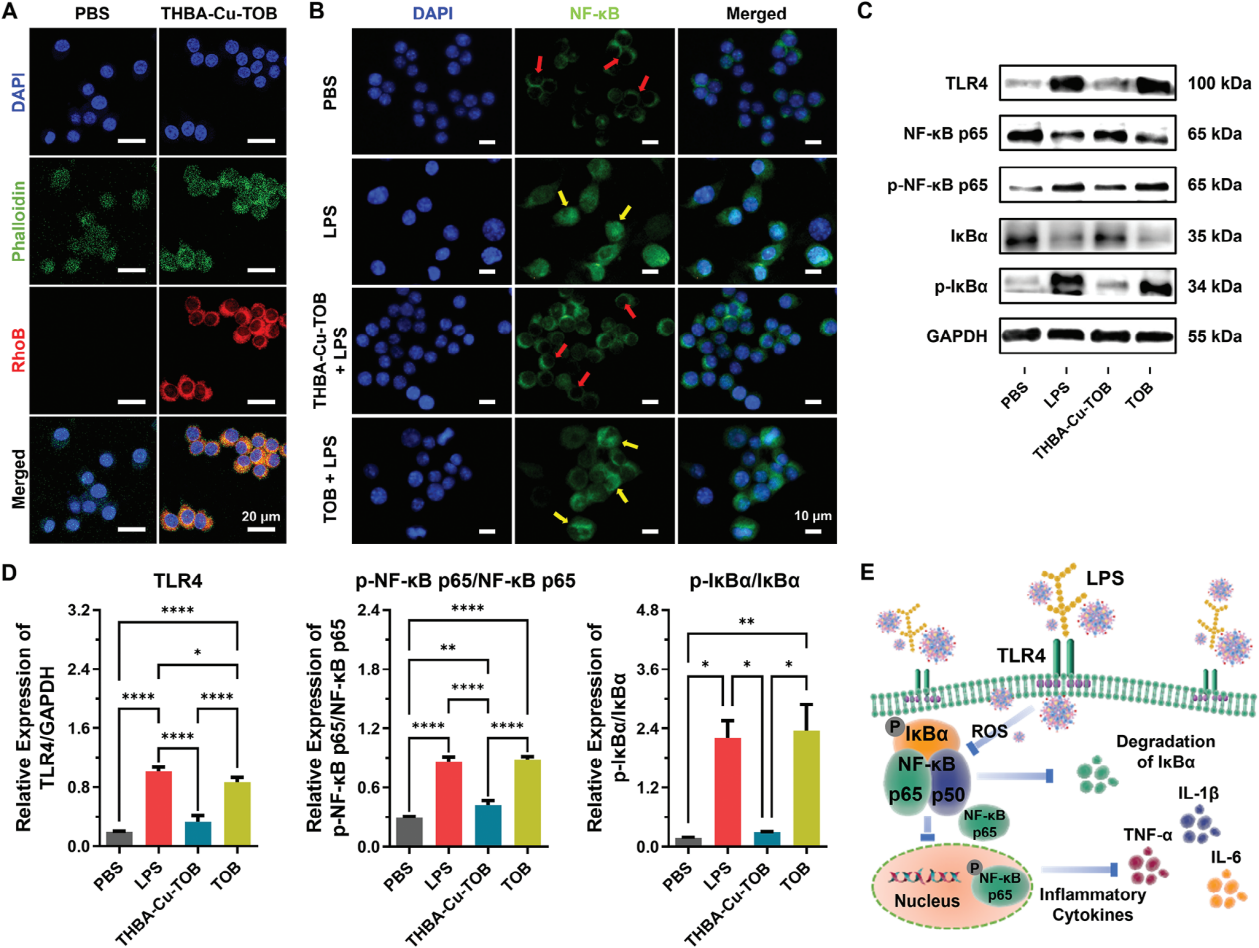
Effect of THBA‐Cu‐TOB NPs on anti‐inflammatory signaling pathway. A) Representative fluorescence images of concentration‐dependent cellular uptake of THBA‐Cu‐TOB@RhoB by RAW264.7 cells. Phalloidin: green; DAPI: blue; RhoB: red. B) Fluorescence images of RAW264.7 cells treated by PBS, LPS, THBA‐Cu‐TOB + LPS, and TOB + LPS, respectively. Blue: DAPI; NF‐κB: green. Red arrows: NF‐κB in the cytoplasm; Yellow arrows: NF‐κB in the cytoplasm and nucleus. C) Western blotting analysis of TLR4, p‐IκBα, p‐NF‐κB‐p65, and GAPDH protein expressions (LPS incubated for 24 h) in RAW264.7 cells and D) corresponding statistical analysis. Data were presented as means ± SD (*n* = 3): not significant (ns), *p* > 0.05, *p < 0.05, **p < 0.01, ***p < 0.001, and ****p < 0.0001. E) Schematic diagram of THBA‐Cu‐TOB NPs in inhibiting TLR4/NF‐κB signaling pathway.

The THBA‐Cu‐TOB NPs on the expression of inflammatory factors produced by macrophage and the related molecular mechanisms were explored at cellular level. As shown in **Figure** **10**A, the cellular internalization of THBA‐Cu‐TOB NPs by RAW264.7 cells was first investigated after RhoB labeling. There was a significant overlap between red fluorescence of nanomaterials and green fluorescence of cell membranes after 2 h incubation, indicating the fusion of nanomaterials and cells. The large endocytosis of THBA‐Cu‐TOB NPs by inflammatory cells provided a basis for immune regulation. In the treatment of infectious corneal ulcers, bacteria, and bacterial biofilm were distributed outside the corneal epithelial cells and partially penetrated into the corneal stroma layer. Immune cells were mainly transported to the lesion site through the corneal stroma layer for sterilization. Therefore, THBA‐Cu‐TOB NPs were administered in the form of eye drops to the ocular surface, which could achieve the effect of “internal response and external combination” in the battle to kill bacteria. When nanomedicines converged with immune cells, oxidative stress and inflammatory response were reduced through immune regulation, promoting the programmed transformation of corneal ulcer wounds into healing.

In the process of immune regulation, it is believed that LPS‐activated TLR4 activated nuclear factor kappa‐B (NF‐κB) pathway to promote transcription and expression of inflammatory factors.^[^ ^13^ ^]^ Specifically, the activated NF‐κB was translocated into the macrophage nucleus and bound to the associated DNA motif to induce transcription of target genes.^[^ ^47^ ^]^ As the receptor of LPS, TLR4 protein expression on the cell membrane surface would significantly increases under the stimulation of LPS, further activating the NF‐κB inflammatory pathway. As shown in Figure 10B, the content of NF‐κB stained with green fluorescence in the control and THBA‐Cu‐TOB NPs treated groups was very low and mainly distributed surrounding the nucleus, indicating that the NF‐κB inflammatory pathway was not activated. Nevertheless, the nucleus stimulated by LPS was almost filled with green fluorescence, showing that a large amount of NF‐κB entered the nucleus and the inflammatory pathway was significantly activated. Meanwhile, there was still a large amount of green fluorescence in the nucleus of the TOB treated group, displaying the lack of anti‐inflammatory effect of free TOB. Overall, it is clearly demonstrated that the component THBA in THBA‐Cu‐TOB NPs inhibited the inflammatory pathway of inflammatory cells (Figure S19, Supporting Information).

NF‐κB is generally inactively latent in the cytoplasm of macrophages and forms trimer p50‐p65‐IκB with inhibitory factor IκBα. When pathogen LPS stimulated macrophages to phosphorylate IκBα, the IκBα will degrade by proteasomes to release NF‐κB. Then, p‐NF‐κB p65 entered the nucleus from the cytoplasm, bound to nuclear DNA to initiate gene transcription, and induced the release to inflammatory mediators. Usually, the expression of TLR4 protein and phosphorylation levels of NF‐κB p65 and IκBα are low in normal macrophages. Under LPS stimulation, the expression of TLR4 protein greatly increased, leading to the increased phosphorylation level of IκBα and NF‐κB p65. Therefore, western blotting analysis was also implemented to quantitatively analyze the expressions of TLR4, NF‐κB p65, and IκBα proteins and their phosphorylation (Figure 10C,D). It was also found that the TOB treatment hardly altered the level of NF‐κB inflammatory pathway activation. In contrast, the expression of TLR4 protein and the phosphorylation of NF‐κB p65, and IκBα significantly decreased in the THBA‐Cu‐TOB NPs treated group. It was verified that the positively charged THBA‐Cu‐TOB NPs could adhere to the negatively‐charged bacterial membrane via electrostatic interaction. Similarly, the positively charged NPs bound onto the surface of bacterial membrane also interacted with the negatively charged LPS containing multiple phosphate groups to block the binding of LPS to TLR4 receptor, further inhibiting the activation of macrophages by LPS (Figure S20, Supporting Information).^[^ ^12^ ^]^ Hence, it could be concluded that THBA‐Cu‐TOB NPs acted in the TLR4/NF‐κB signaling pathway by rapidly killing bacteria to reduce LPS secretion and blocking the binding of LPS to TLR‐4 protein, ultimately reducing corneal inflammatory response (Figure 10E).

In the clinical treatment guidelines for corneal infections, it is explicitly stated that hormone based anti‐inflammatory drugs can only be used to promote wound repair after the symptoms of infection have been significantly reduced, because the bacteria have strong invasiveness and can lead to corneal perforation and extensive ulcers within 2–3 days after infection. In the early stage of clinical treatment, empirical medication is generally used and pathological identification is carried out. This process becomes indispensable in relying on the patient's own immune response to actively sterilize. Importantly, free eye drops have a short retention time on the ocular surface and low permeability to the corneal physiological barrier, resulting in extremely low bioavailability of the drug (less than 5%).^[^ ^48^ ^]^ Moreover, frequent drops of free eye drops can also cause eye surface irritation. Significantly, THBA‐Cu‐TOB NPs achieved its retention on the ocular surface and physiological barrier penetration based on electrostatic interaction with the mucin layer. The reversible chemical bonds formed between TOB and other components in THBA‐Cu‐TOB significantly improved the stability avoiding path loss of drugs. Furthermore, the excellent penetrability of THBA‐Cu‐TOB NPs into biofilm and corneal physiological barrier as well as the pH responsive release to infected environment greatly enhances the local concentration of drug in bacteria elimination. Simultaneously, the released THBA and Cu^2+^ can eliminate ROS, reduce inflammation, and promote corneal epithelial cell migration for corneal ulcer repair. Therefore, the modular nano functional material system is a completely different treatment model from clinical guidelines.

## 3 Conclusions

In summary, we proposed a simple one‐step strategy for the rapid and green preparation of modular THBA‐Cu‐TOB NPs based on chemical bonds response to pH. The loading of TOB increased the bonding between components, thus improving the stability of nanomaterials to avoid drug path loss. THBA‐Cu‐TOB NPs achieved local high concentration release through cleavage of acid responsive chemical bonds in the infected area. The THBA‐Cu‐TOB NPs penetrated the entire 20 µm thick biofilm based on the cationic charges and nano size effect, achieving bacteria elimination within the biofilm. Notably, the equivalent TOB concentration in THBA‐Cu‐TOB NPs was only 1.6% of the free TOB with the same sterilization efficiency. In the infectious corneal ulcer animal model, positively charged THBA‐Cu‐TOB NPs significantly improved the retention on the ocular surface and endocytosis by HCECs. After treatment, the corneas treated by THBA‐Cu‐TOB NPs basically returned to the normal corneal structure and morphology in infectious corneal ulcer healing, which could be attributed to the modular design of the nanomaterial system, including release of TOB to achieve sterilization, ROS elimination and anti‐inflammatory by THBA, and promotion migration of corneal epithelial cell by Cu^2+^.

## 4 Experimental Section

### Materials and Reagents

3,4,5‐trihydroxybenzaldehyde (THBA, 98%), Cu^2+^ standard solution (1 mg mL^−1^), tobramycin (TOB, 98%), 3,3′‐diethyloxacarbocyanine iodide (DiOC_2_(3), 98%), carbonyl cyanide 3‐chlorophenylhydrazone (CCCP, 98%), aniline blue (AB, water‐soluble), mucin from bovine submaxillary gland, and rhodamine B (RhoB) isothiocyanate were purchased from Shanghai Makclin Biochemical Co., Ltd. ε‐poly‐ʟ‐lysine (EPL, 94%) was obtained from Zhengzhou Bainafo Bioengineering Co., Ltd. 2′,7′‐dichlorodihydrofluorescein diacetate (DCFH‐DA, 97%) was obtained from Sigma Aldrich. Sulfo‐cyanine7 (Cy7) NHS ester (5 mg, 95%) was purchased from Aladdin Biochemical Technology Co., Ltd. *P. aeruginosa* (ATCC 15 442) was obtained from Guangdong Microbial Culture Collection Center (GDMCC). LIVE/DEAD Viability/Cytotoxicity Kit and LIVE/DEAD *Bac*Light Bacterial Viability Kit were purchased from Thermo Fisher. Conventional reagents and solvents were obtained without additional purification.

### Preparation of NPs

First, Cu^2+^ solution (250 µL) was added into a round bottom flask with a mixture of ethanol and water (C_2_H_5_OH: H_2_O = 24: 1 v/v, 10 mL), and pH of the mixture was adjusted to 10 using sodium hydroxide solution (NaOH, 1 m) under stirring. Second, THBA (100 µL, 100 mg mL^−1^), EPL (100 µL, 10 mg mL^−1^), and TOB (100 µL; 0, 0.5, 1.0, 1.5, and 2.0 mg mL^−1^) were added and stirred at 60 °C for 2 h (800 rpm). After that, the solution was centrifuged to collect the sediment and washed three times. Finally, the brown precipitate was dispersed into water again, treated with ultrasound for 1 min and stored away from light for later use (THBA‐Cu NPs: 1 mg mL^−1^; THBA‐Cu‐TOB NPs: 1 mg mL^−1^).

### Minimum Inhibitory Concentration (MIC) and Minimum Bactericidal Concentration (MBC) Assay

First of all, *P. aeruginosa*, separately stored in −80 °C refrigerator, was oscillated overnight in 5 mL tryptic soy broth (TSB) medium. Then, *P. aeruginosa* cultured overnight was taken and incubated again in 5 mL TSB medium. Then, 1 mL of bacterial suspension in the logarithmic phase was centrifuged, washed with PBS three times, and suspended in 1 mL sterile PBS for later use. Briefly, a series of NPs solutions (100 µL) were prepared in a 96‐well plate using the double dilution method. Then, 100 µL of *P. aeruginosa* suspension was added to each well and incubated in a 37 °C shaker for 24 h. The clear and transparent wells with the lowest concentration were selected as MIC after visual observation. 10 µL suspension from each well was dropped onto Luria‐Bertani (LB) agar plates and incubated for 24 h in a 37 °C incubator, and the concentration of the solution in the well without *P. aeruginosa* colony was selected as MBC.

### pH‐Responsive Release of Cu^2+^ and TOB Evaluation

*1) Cu^2+^ Release*: 150 µL NPs suspension (1 mg mL^−1^) was transferred into a dialysis tube (MWCO: 500–1000 Da) and then wholly absorbed into a 50 mL centrifuge tube prefilled with 15 mL of PBS or water. 1 mL of dialysis solution was taken at different times and ICP‐MS test was performed centrally. *Note: 1 mL PBS was added to keep the total volume unchanged. 2) TOB Release*: Under acidic conditions, the sulfonate group of aniline blue and the amino group of TOB formed a complex by electrostatic interaction, resulting in the shift of the red maximum absorption peak of aniline blue to 692 nm, with an apparent fading peak at 606 nm. The change of absorbance value at the absorption peak and fading peak was proportional to TOB concentration. The 606 nm was chosen as the determination wavelength and the fading spectrophotometry was established to determine the release amount of TOB. First, the standard curves of TOB with different concentrations were established to obtain the linear regression equation. Second, THBA‐Cu NPs was taken as the control to test the TOB content in different THBA‐Cu‐TOB NPs. Finally, TOB amount release from THBA‐Cu‐TOB NPs in dialysate was tested at different pH. *3) TEM Observation*: NPs were dispersed in PBS at different pH and incubated on a shaker for 2 h. Then, 10 µL PBS was dropped onto a copper mesh and dried naturally for TEM observation.

### In Vitro Antibacterial Experiments

Under different pH values, *P. aeruginosa* suspensions (900 µL, 10^8^ CFU mL^−1^) were added with 100 µL of NPs (the final concentration was 8, 16, and 32 µg mL^−1^). The solutions were ultrasonically treated for 1 min and incubated on a 37 °C shaker for 2 h. A series of diluents with a dilution ratio of 10^n^ (*n* = 2, 3, 4, and 5) were prepared and 10 µL of the solution was taken and incubated in LB agar plates for 16 h. The number of colonies that could be clearly distinguished was recorded as C. Finally, the bacteria count in the original suspension was C×10^n+2^ CFU mL^−1^ and the antibacterial rate (%) = [(lg CFU_control_ – lg CFU_NPs_)/lg CFU_control_] × 100%. After collecting the complex of NPs interacting with *P. aeruginosa*, the bacterial activity was observed by CLSM via Live/Dead staining.

### Detection of Membrane Potential of P. aeruginosa

DiOC_2_(3) was used for analyzing membrane potentials of bacteria.^[^ ^49^ ^]^ The *P. aeruginosa* solutions at the logarithmic growth stage (OD = 0.5–0.7) were diluted to 10^6^ CFU mL^−1^ with sterile PBS buffer before NPs adding. CCCP was used to destroy mitochondrial membrane potential by removing proton gradient, resulting in a decrease in red fluorescence intensity and a decrease in red/green fluorescence signal ratio. CCCP‐untreated bacteria and CCCP‐treated bacteria were used as a negative and positive control groups, respectively (CCCP: 5 µm; *P. aeruginosa*: 10^6^ CFU mL^−1^). After incubation for 1 h, 10 µL DiOC_2_(3) working solution (3 mm) was added and incubated in the dark for 30 min. Finally, the relative red and green fluorescence intensities of all groups were analyzed by flow cytometer (excitation at 488 nm; emission in both red‐630 nm and green‐515 nm channels).

### Zeta Potential of NPs‐treated Bacterial Cells and LPS

The zeta potential of the *P. aeruginosa* suspension (10^6^ CFU mL^−1^) was measured after being treated by EPL, TOB, THBA‐Cu, and THBA‐Cu‐TOB, respectively. Zeta potential of the initial LPS solution (5 µg mL^−1^) and THBA‐Cu‐TOB NPs treated sample were also measured.

### SEM and TEM Assay

*1) SEM Observation*: The NPs dispersion (50 µL, 1 mg mL^−1^) was incubated with *P. aeruginosa* suspension (950 µL, 10^8^ CFU mL^−1^) at pH 7.4 and 5.5 in the shake for 2 h (37 °C, 40 rpm). After removing the liquid via centrifugation, the NPs‐bacterial complex was obtained and refrigerated overnight after adding 100 µL of glutaraldehyde. The fixative was removed and washed lightly twice with PBS followed by gradient dehydration. Finally, the NPs‐bacterial complex was placed on a clean silicon surface to put at a position with natural ventilation for gold spraying before SEM observation. *2) TEM Observation*: Following the above steps, the gradient dehydrated NPs‐bacterial complex was placed on molybdenum net and dried at room temperature for TEM observation.

### Biofilm Penetrated and Elimination Assay

According to the previous work, various nanomaterials were labeled with RhoB or Cy7. Briefly, overnight incubation of *P. aeruginosa* suspension (100 µL) was added to 5 mL of fresh TSB medium for further 4 h incubation. Then, 10 µL of logarithmic *P. aeruginosa* suspension was added to a glass bottom cell culture dish (Φ 15 mm, Thermo Scientific) with 2 mL TSB medium and incubated for 3 days at 37 °C to form biofilm. *Note: the culture medium (2 mL) was changed daily and the entire process was handled lightly to prevent damage to the biofilm*. After that, the suspension was sucked from the Petri dishes and washed twice with PBS. Then, PBS, THBA‐Cu@RhoB, THBA‐Cu‐TOB@RhoB, TOB@RhoB, and EPL@RhoB were added separately to the dish. After incubation at 37 °C for 2 h, the biofilms were slightly washed with PBS twice to remove non‐adhesive materials. After that, 50 µL LIVE/DEAD Bacterial Viability Kit was added for 20 min incubation, and 50 µL DAPI was added for 10 min incubation in the dark. The biofilms were rinsed three times with PBS and photographed under CLSM. In the biofilm elimination test, PBS, THBA‐Cu NPs, THBA‐Cu‐TOB NPs, TOB (40, 80, and 160 µg mL^−1^), and EPL were added separately. After being incubated at 37 °C for 2 h, the biofilms were washed gently with PBS three times. The fluorescent photos were taken by CLSM after being stained by LIVE/DEAD Bacterial Viability Kit at room temperature.

### Anti‐Inflammatory Effect

*1) LPS‐Stimulated Macrophages*:^[^ ^42^ ^]^ First, RAW264.7 single cell suspension at logarithmic growth stage were inoculated into 24 well culture plates (1.5×10^5^ cells per well) and cultured in incubators for 24 h. The medium was sucked out. 1 mL of LPS‐containing medium (1 µg mL^−1^) was added in each well of the LPS‐stimulated group and cultured for 2, 12, and 24 h, respectively. 1 mL of LPS‐free medium was added in each well in the blank control group. Second, untreated and LPS stimulated cells were used as negative and positive control groups, respectively. The cell activity of NPs was evaluated after being incubated with LPS at different concentrations for 24 h. Finally, cells in the control, LPS and LPS+NPs groups were rinsed and treated with DCFH‐DA (10 µm) in the dark for 30 min. After washing with PBS three times, the average DCF fluorescence intensity generated by the intracellular ROS of collected cells was quantitatively measured via flow cytometer and analyzed by FlowJo software. *2) H_2_O_2_‐Stimulated Macrophages*:^[^ ^50^ ^]^ RAW264.7 cells were seeded in a 24 well plate (1.5×10^5^ cells per well) overnight to allow cell attachment. NPs were added into the plate for 24 h incubation after the macrophages were pretreated with H_2_O_2_ (50 µm) for 2 h. H_2_O_2_ treatment was used as a positive control group. DCFH‐DA was diluted to a final concentration of 10 µM with a serum‐free medium. After the medium was sucked out of the plate, 500 µL of culture medium was added to each well and incubated at 37 °C for 25 min in the dark. Then, DAPI was added for further 5 min incubation. After incubation, observation was taken under an inverted fluorescence microscope after three times PBS washing. *3) Inflammatory response*: LPS (1 µg mL^−1^) pretreated macrophages (2 h) and NPs (pH 7.4 and 5.5) were incubated for 24 h. qPCR analysis was used for detecting mRNA expressions of inflammatory factors (TNF‐α, IL‐6, and IL‐1β).

### Cell Migration Assay

Cell migration was evaluated by scratch test. Briefly, 10^5^ HCECs per well were inoculated on 6 well plates and starved in FBS‐free medium for 24 h. Next, the HCECs layer was scratched with the tip of a 200 µL pipette. The floating cells and debris were washed away with PBS before nuclei staining by Hoechst 33 258. After that, the HCECs were treated by PBS (control), Cu^2+^, THBA, and NPs (pH 7.4 and 5.5), respectively. The difference of wound area at 0 and 24 h was observed by the blue fluorescence channel under fluorescence microscope (excitation: 360 nm, emission: 450 nm) to determine the degree of scratch closure. The migration rate was analyzed by using Image J software. Cell migration (%) = [(M_scratch area 0 h_ – M_scratch area 24 h_)/M_scratch area 0 h_] × 100%.

### Ocular Surface Retention and Corneal Penetration of NPs

*1) Corneal Surface Retention*: THBA‐Cu@Cy7 and THBA‐Cu‐TOB@Cy7 NPs were dropped into anesthetized rat's right eye twice with 5 µL each time at an interval of 1 min. *Note: manually blink rat's eyes 10 times to promote even distribution of the drops on the eye surface*. The fluorescence images and the fluorescence intensity were recorded at different periods (excitation: 747 nm, emission: 774 nm). Retention efficiency (%): = [F_n h_/F_0 h_] × 100%. *2) Corneal Penetration*: THBA‐Cu@RhoB and THBA‐Cu‐TOB@RhoB NPs were dropped onto rat corneal surface, shielded from light for 1 h, and the rats were euthanized to take the corneas. Then, the corneas were cleaned with PBS and observed using a microscope.^[^ ^45^ ^]^

### Aeruginosa Infection Model of Corneal Trauma

Healthy male Sprague‐Dawley (SD) rats (weight: 160–180 g, age: 6–8 weeks) were raised in the Laboratory Animal Centre of Wenzhou Medical University for in vivo evaluation. All experimental animal procedures were approved by the Institutional Animal Care and Use Committee of Wenzhou Medical University (China, SPF Level, SYXK 2018‐0017). A circular area with a diameter of 5 mm was labeled in the central cornea of rats under the microscope, and the upper cortex of the labeled area was scraped with a needle of a sterilized 1 mL syringe. *P. aeruginosa* suspension was dropped to the corneal wounds of rats twice (5 µL, 10^8^ CFU mL^−1^) at an interval of 5 min. Then, *sixty* experimental rats were randomly divided into 5 groups, with 12 rats in each group. *Note: NPs (50 µL, 0.5 mg mL* ^−1^ *) and TOB (50 µL, 3 mg mL* ^−1^ *) were packaged in solution form in a 10 mL medicinal eye drops bottle for use*. In the control group, the cornea of rats was not treated. In the infected groups, the sterilized PBS was added to the corneal wounds of rats on days 1–3 and 5–6 (1 drop per day in the morning, middle, and evening) and days 8–11 (1 drop per day at noon). In NPs treated groups, THBA‐Cu and THBA‐Cu‐TOB NPs eye drops were added to the corneal wounds of rats on days 1–3 and 5–6 (1 drop per day in the morning, middle, and evening) and days 8–11 (1 drop per day at noon). In the TOB group, the TOB eye drops were added to the corneal wounds of rats on days 1–3 and 5–6 (1 drop per day in the morning, middle, and evening) and days 8–11 (1 drop per day at noon).

At day 1, 4, 7, and 12, corneal infection and pathological characteristics were observed under a slit‐lamp microscope and photographed. The severity of bacterial keratitis was scored according to corneal opacity area, density, and surface irregularity. The body weight of rats in each group was measured at day 1, 4, 7, and 12 of the treatment periods. At day 12, all rats were anesthetized and sacrificed. Their eyeballs were extruded by pinching the two temporals by hands and quickly removed by clamping the bottom with curved tweezers. The eye tissue from each group was directly immersed in sterilized PBS, and subjected to ultrasonic treatment for 10 min. Then, the bacteria content in the suspension was counted by tenfold dilution plate colony counting method. 3 eyeballs from each group were cleaned with normal saline and soaked in eye fixative solution at room temperature for 24 h. The corneas were cut along the corneal limbus with tissue scissors under a microscope. Subsequently, corneal tissue was stained by Gram staining solution to observe the inside bacterial clearance. Corneal tissue in each group was stained with H&E to observe the cell infiltration and tissue necrosis, and corneal tissue was also stained with immunofluorescence to detect the expression of inflammatory factors. The remaining fresh corneal tissues were placed in a tube and frozen in liquid nitrogen. The expression levels of inflammatory factors in each group were detected by qPCR and western blotting.

### TLR4/NF‐κB Signaling Pathway

TLR4/NF‐κB signaling pathway played an essential role in oxidative stress, inflammation, and apoptosis. *1) Cellular Internalization*: Cell uptake of the THBA‐Cu‐TOB NPs was detected by fluorescence imaging. Briefly, RAW264.7 cells (1 × 10^5^ cells per well) were incubated with THBA‐Cu‐TOB@RhoB NPs (40 µg mL^−1^) for 2 h. After that, cells were stained successively with phalloidin‐FITC (green, C1033, Beyotime) and DAPI (Blue, C1005, Beyotime). Before dying, the cells on glasses were rinsed and covered with an antifade polyvinylpyrrolidone mounting medium (P10232, Beyotime). Finally, the endocytosis behavior of macrophages to the THBA‐Cu‐TOB NPs was observed and analyzed by CLSM. *2) Immunofluorescence Evaluation*: RAW264.7 cells (1 × 10^5^ cells per well) were cultured overnight. Cells were pre‐stimulated by LPS (1 µg mL^−1^) for 2 h, followed by incubation with THBA‐Cu‐TOB NPs (40 µg mL^−1^) and TOB (50 µL, 3 mg mL^−1^) for 24 h, respectively. After that, the cells were fixed with 4% paraformaldehyde for 15 min, permeabilized with 0.1% Triton X‐100 for 10 min, and blocked with 2% BSA for 45 min at room temperature. The cells were labeled with primary antibody of NF‐κB (1: 100 in 0.1% BSA), incubated at 4 °C overnight, and labeled with FITC‐conjugated secondary antibody (1: 2000 dilution) for 45 min at room temperature. Finally, after sucking the excess liquid with absorbent paper, the stained cells were mounted with an antifade mounting medium with DAPI (Blue, P0131, Beyotime) for images observation under a fluorescence microscope (DMi8, Leica). The activation of NF‐κB in the nucleus was evaluated through green fluorescence observation. *3) Western Blotting Assay*: Macrophages from each group were collected and lysed with RIPA lysis buffer (Beyotime) to obtain protein samples. Next, the proteins (20 µg) were separated by gel electrophoresis (10% SDS‐PAGE), then transferred to a polyvinylidene difluoride (PVDF) membrane (Millipore), and finally sealed at room temperature in TBST + 5% skim milk for 1 h. After that, the membranes were incubated overnight at 4 °C with the following primary antibodies including TLR4, NF‐κB P65, p‐NF‐κB P65, IκBα, p‐IκBα, and GAPDH. Subsequently, the protein blots were visualized with an enhanced chemiluminescence detection kit after incubation with HRP‐conjugated secondary antibody at room temperature for 1 h. Finally, Image J software was used to measure the signal intensity and quantitatively analyze the spectral band.

### Statistical Analysis

All test data were presented as mean ± standard deviation, and the statistical analysis was implemented using the *GraphPad Prism 9* software. Unlike comparing multiple groups by one‐way variance analysis, the statistically significant differences between the two groups were compared by unpaired t‐tests. Additionally, *p* > 0.05 indicated no statistical (ns) significance; *p < 0.05, **p < 0.01, ***p < 0.001, and ****p < 0.0001 indicated a statistically significant difference.

## Conflict of Interest

The authors declare no conflict of interest.

## Supporting information

Supporting Information

## Data Availability

The data that support the findings of this study are available from the corresponding author upon reasonable request.
