# Supplementary material for: An Antibiotic Nanobomb Constructed from pH‐Responsive Chemical Bonds in Metal‐Phenolic Network Nanoparticles for Biofilm Eradication and Corneal Ulcer Healing
Source: Adv Sci (Weinh). 2024 Mar 15;11(22):2309086. doi: 10.1002/advs.202309086 (PMC11165475; doi:10.1002/advs.202309086)
Supplement: Supplementary file 1 — Supporting Information [file ADVS-11-2309086-s001.pdf]

ADVANCED SCIENCE
Open Access

Supporting Information

for *Adv. Sci.*, DOI 10.1002/advs.202309086

An Antibiotic Nanobomb Constructed from pH-Responsive Chemical Bonds in Metal-Phenolic Network Nanoparticles for Biofilm Eradication and Corneal Ulcer Healing

Qiang Gao, Xiaoying Chu, Jie Yang, Yishun Guo, Hanwen Guo, Siyuan Qian, Ying-Wei Yang and Bailiang Wang**

Supporting Information

An Antibiotic Delivery Constructed from pH-Responsive Chemical Bonds in Metal-Phenolic Network Nanoparticles for Biofilm Eradication and Corneal Ulcer Healing

Qiang Gao, Xiaoying Chu, Jie Yang, Yishun Guo, Hanwen Guo, Siyuan Qian, Ying-Wei Yang,* and Bailiang Wang*

Q. Gao, X. Chu, Y. Guo, H. Guo, S. Qian, Prof. B. Wang
National Engineering Research Center of Ophthalmology and Optometry, Eye Hospital, Wenzhou Medical University, Wenzhou 325000, China
E-mail: blwang@wmu.edu.cn

Prof. Y. Yang
College of Chemistry, Jilin University, 2699 Qianjin Street, Changchun 130012, P. R. China
E-mail: ywyang@jlu.edu.cn

Q. Gao, Prof. B. Wang
State Key Laboratory of Ophthalmology, Optometry and Visual Science, Wenzhou Medical University, Wenzhou, 325027, China

Prof. B. Wang
NMPA Key Laboratory for Clinical Research and Evaluation of Medical Devices and Drug for Ophthalmic Diseases, Wenzhou, 325027, China

Dr. J. Yang
School of Life Sciences, Jilin University, 2699 Qianjin Street, Changchun 130012, P. R. China

Experimental Section

***Characterization of NPs*:** The size and zeta potential of nanomaterials were measured using dynamic light scattering (DLS) equipment by Zetasizer Nano-ZS from Malvern Instruments. The absorption spectra of NPs were measured by ultraviolet-visible (UV-vis) absorption spectrometry (Agilent CARY5000, USA). The NPs-bacteria interaction was observed and analyzed by field-emission scanning electron microscopy (FE-SEM, HITACHI SU8010, Japan) and high-angle annular dark-field

scanning transmission electron microscope (HAADF-STEM, FEI Talos™ F200S 200 kV, USA). The chemical composition and structure of NPs were analyzed by X-ray photoelectron spectroscopy (XPS, Kratos AXIS Ultra DLD, UK) and Fourier transform-infrared spectroscopy (FT-IR, Bruker Tensor II, German). The release of Cu^{2+} from NPs in the solution was measured by inductively coupled plasma mass spectrometry (ICP-MS, Agilent 7850, USA). The optical density (OD) value was measured by a microplate reader (Thermo Fisher Varioskan LUX, USA). The retention time of NPs on the ocular surface was estimated by a multimode optical in vivo imaging system (PerkinElmer IVIS Lumina XRMS Series III, USA). The fluorescence spectra and pathological section results were assessed by an upright/inverted fluorescence microscope (DM4 B/DMi8, Leica, Germany). The results of biofilm penetration and eradication of NPs were obtained by confocal laser scanning microscopy (CLSM, Zeiss LSM 880 Ariyscan, Germany). The bacteria and cells were identified automatically by flow cytometry (Beckman Coulter CytoFLEX, USA). The images of bacterial colonies were captured with a Nikon camera and colored with Photoshop.

Dissipative Particle Dynamics (DPD) Simulation**:** The self-assembly process of EPL, THBA, TOB, and Cu^{2+} in solution was simulated by the DPD method. In DPD, a box size of 15×15×15 nm^3 with a three-dimensional periodic boundary was constructed. We synthesized several atoms or groups of atoms into a bead, reducing the degree of freedom in each molecule by interacting with the particles through an effective force field. The details of bond length and bond angle in the compound structure were ignored in the simulation method. The temperature was set at 298 K, the time step was 10 fs, the bead force field was martini,[S1] and the total simulation time of the self-assembly process was 20 ns.

Cell Lines and Cell Cultures**:** Human corneal epithelial cells (HCECs), human umbilical vein endothelial cells (HUVECs), and mouse fibroblasts cells (L929 cells) were purchased from American Type Culture Collection (ATCC). HCECs, HUVECs, and L929 cells were incubated in 25 cm^2 cell culture flasks (Corning) and cultured in DMEM/F12 (Gibco) and DMEM (Gibco), containing 10% fetal bovine serum (Ausgenex), penicillin (100 U/mL), and streptomycin (100 μg/mL) (Gibco) at 37 °C in

a humidified 5% CO_2 atmosphere. The cells were passaged with 0.25% trypsin containing 0.02% ethylene diamine tetra-acetic acid (EDTA, Gibco) every 3 days. RAW264.7 macrophages were obtained from ATCC and raised in 10 cm^2 dishes, cultured in DMEM medium containing 4.5 g/L D-Glucose (Corning) supplemented with 10% fetal bovine serum (Ausgenex) plus penicillin (100 U/mL) and streptomycin (100 μg/mL) (Gibco). The macrophages were passed every 2 days with cell scrapers.

***Tissue Sections and Staining*:**[S2] Corneal tissues were fixed with 4% paraformaldehyde fixative at room temperature for 24 hours and dehydrated overnight with an automatic dehydrator. The paraffin-embedded corneal tissues were sectioned and stained with hematoxylin and eosin (H&E), covered with a cover glass, and dried naturally. The sections were placed under a fluorescence microscope (DM4 B, Leica) to photograph the healing of corneal ulcers and the infiltration of inflammatory cells.

***Immunofluorescent Staining*:**[S2] After the sections of corneal tissue were dewaxed, goat serum blocking solution was dropped at 37 °C for 1 h. Then, the primary antibody (anti-TNF-α, IL-6, and IL-1β antibody: 1:100) was added, and the sections were incubated in the wet box overnight at 4 °C. Subsequently, the sections were incubated for 60 min in a wet box at room temperature after dropping fluorescence-labeled secondary antibody. After being cleaned, DAPI staining was employed to seal the sections, and the staining results were observed and photographed under a fluorescence microscope (DM4 B, Leica).

***RNA Isolation, cDNA Synthesis, and Real-Time Quantitative Polymerase Chain Reaction (qPCR) Analysis*:**[S3] RNA from macrophages or corneal tissues was extracted by Trizol (Invitrogen, Carlsbad, CA, USA) method and its concentration was measured by NanoDrop 2000 (Thermo Scientific). 1 μg RNA was taken from each sample and cDNA was synthesized by reverse transcription via enzymatic reaction using PrimeScript™ RT reagent Kit (RR037A, TaKaRa). qPCR analysis was performed by TB Green® Premix Ex Taq™ II reagents (RR802A, TaKaRa, Japan) and conducted using a 7500 Fast Real-Time PCR System (Applied Biosystems): Holding Stage: 95 °C for 30 s. Cycling Stage: 40 cycles at 95 °C for 3 seconds, 60 °C for 30 s. Quantitative analysis was performed using the comparative (ΔΔCT) method and each assay was

normalized by amplifying the housekeeping cDNA R-GAPDH. Table S1 listed the cytokines and sequences of forward and reverse primers.

***Western Blotting Analysis*:**[S3] RAW264.7 cells or corneal tissues were cleaned with PBS. In an ice bath, the protein was extracted with RIPA lysis buffer (P0013B, Beyotime) containing 1 mM phenylmethanesulfonyl fluoride (PMSF). After being treated by low temperature and high-speed centrifugation (4 °C, 25 min, 15000 rpm), the supernatant, namely the total protein extract, was resulted and measured by Pierce™ BCA Protein Assay Kit (23225, Thermo Scientific). 30 μg of protein sample was added to 10% SDS-Page gel and transferred to PVDF membrane (IPVH00010, Immobilon). After being sealed with 5% BSA for 1 h, an appropriate diluted primary antibody was added to incubate overnight at 4 °C. Then, the secondary antibody was added and incubated at room temperature in the dark for 1 h. The specific protein bands were displayed using an ultra-sensitive ECL chemiluminescence substrate (A38555, Thermo Scientific) and imaged using Bio-Rad ChemiDoc MP, a commercial imaging system. Image intensity was calculated using ImageJ software. β-Actin and GAPDH were used as internal reference proteins. All antibodies are listed in Table S2.

***In Vitro Cytotoxicity Evaluation: 1) CCK-8 Assay*:** The cytotoxicity of THBA-Cu and THBA-Cu-TOB NPs was detected by the CCK-8 method. Briefly, mouse fibroblast cells (L929 cells, 10^4 cells/well) and human corneal epithelial cells (HCECs, 10^4 cells/well) were seeded onto 96-well plates, respectively. Then, various concentrations of NPs (10, 20, 40, 80, and 160 μg/mL) at pH 7.4 and 5.5 were added and co-cultured with cells for 24 h, respectively. After that, CCK-8 solution (10%, 10 μL) was added into each well and incubated for 1 h. The optical density (OD) value of each well was measured at 450 nm using a microplate Bio-Rad reader. Cell viability (%) = $[(OD_{NPs} - OD_{control})/OD_{control}] \times 100\%$, data were presented as mean ± standard deviation (SD) (n = 5). ***2) Hemolysis Assay*:** A hemolysis test was used to detect the rupture of red blood cells. For positive control of the hemolysis test, we added an equal volume of distilled water into red blood cell suspension to trigger low permeability hemolysis. Alternatively, we chose a solution that destroys the red cell membrane, such as 1% Triton-X 100, which was able to remove proteins from the red cell membrane and destroy red blood cells in a relatively short time. In the negative control experiment,

erythrocytes were placed in a PBS buffer solution (pH 7.4) to maintain the isotonic environment required by erythrocytes. The cationic NPs were added into red blood cell suspension and co-incubated at 37 °C for 2 h, and then the mixture was centrifuged at 1000 rpm for 15 min to get supernatant with an absorbance value of 545 nm. Hemolysis (%) = $[(OD_{NPs} - OD_{PBS})/(OD_{Triton\text{-}X} - OD_{PBS})] \times 100\%$, data were presented as mean ± standard deviation (SD) (n = 5).

In Vivo Safety Evaluation**:** Six rats were randomly divided into two groups and treated with one drop of sterilized PBS (control) and THBA-Cu-TOB NPs eye drops in the morning, afternoon, and evening (right eye), respectively, for 7 days. Rats' blood was collected after anesthesia on day 8 to perform routine blood examination that mainly included blood cell examination, covering white blood cell (WBC), red blood cell (RBC), hemoglobin (HGB), mean corpuscular volume (MCV), mean corpuscular hemoglobin (MCH), mean corpuscular hemoglobin concentration (MCHC), and platelets (PLT), to determine occurrence of infection, anemia, hematological diseases or even platelet diseases. The biochemical examination was mainly used for checking liver and kidney blood biochemical indicators, including aspartate aminotransferase (AST), alanine aminotransferase (ALT), urea (UREA), and creatinine (CREA), for the detection of the viscera diseases and abnormalities. Finally, all rats were anesthetized and sacrificed for toxicological examination of the main organs, including the cornea, heart, liver, spleen, lung, and kidney. The biosafety of the THBA-Cu-TOB NPs in vivo was comprehensively evaluated by the above indicators and detection analysis. The clinical scoring standard refers to Table S4.

Table S1. Primers used in qPCR.

Target (Rat)	Forward Primer	Reverse Primer
TNF-α	CCACGCTCTTCTGTCTACTGA	GATGATCTGAGTGTGAGGGTCT
IL-1β	ATGAGGACCCAAGCACCTTC	AGCTCACATGGGTCAGACAG
IL-6	CTGGTCTTCTGGAGTTCCGTT	GGTCTTGGTCCTTAGCCACTC
GAPDH	GAAGCTGGTCATCAACGGGA	GAAGGGGCGGAGATGATGAC

Table S2. Antibodies for western blotting and immunofluorescent staining.

Antibody	Company	Category Number	Examination Type	Dilution
		**Primary Antibody**		
Rabbit anti-TLR4	CST	14358S	Western Blotting	1:1000
Rabbit anti-NF-κB p65	CST	8242S	Western Blotting<br>Immunofluorescent Staining	1:1000
Rabbit anti-p-NF-κB p65	CST	3033S	Western Blotting	1:1000
Rabbit anti-IκBα	CST	4812S	Western Blotting	1:1000
Rabbit anti-p-IκBα	CST	2859S	Western Blotting	1:1000
Rabbit anti-GAPDH	CST	5174S	Western Blotting	1:1000
Mouse anti-β-actin	CST	3700S	Western Blotting	1:1000
Rabbit anti-IL-1β	CST	12703S	Western Blotting<br>Immunofluorescent Staining	1:2000<br>1:500
Mouse anti-IL-6	Abcam	ab9324	Western Blotting	1:2000
Rabbit anti-TNF-α	Abcam	ab66579	Western Blotting	1:1000
IL-6 rabbit pAb	ZENBIO	500286	Immunofluorescent Staining	1:100
TNF alpha rabbit pAb	ZENBIO	346654	Immunofluorescent Staining	1:100
Goat anti-rabbit IgG H&L (HRP)	Abcam	ab205718	Western Blotting	1:2000
Rabbit anti-mouse IgG H&L (HRP)	Abcam	ab6728	Western Blotting	1:2000
Anti-rabbit IgG (H+L), F(ab')2 Fragment (Alexa Fluor® 488 Conjugate)	CST	4412S	Immunofluorescent Staining	1:2000
Anti-rabbit IgG (H+L), F(ab')2 Fragment (Alexa Fluor® 594 Conjugate)	CST	8889S	Immunofluorescent Staining	1:2000

Goat anti-rabbit IgG H&L (HRP)	Abcam	ab205718	Western Blotting	1:2000
Rabbit anti-mouse IgG H&L (HRP)	Abcam	ab6728	Western Blotting	1:2000
Anti-rabbit IgG (H+L), F(ab')2 Fragment (Alexa Fluor® 488 Conjugate)	CST	4412S	Immunofluorescent Staining	1:2000
Anti-rabbit IgG (H+L), F(ab')2 Fragment (Alexa Fluor® 594 Conjugate)	CST	8889S	Immunofluorescent Staining	1:2000

Table S3. MIC and MBC results of the different materials.

Materials	MIC, μg/mL		MBC, μg/mL	
	pH 6.8	pH 5.5	pH 6.8	pH 5.5
THBA	> 250	> 250	> 250	> 250
Cu^{2+}	> 250	> 250	> 250	> 250
EPL	64	64	128	128
TOB[S4]	1	1	2	2
THBA-Cu NPs	> 250	> 250	> 250	> 250
THBA-Cu-TOB_1 NPs	> 250	> 250	> 250	> 250
THBA-Cu-TOB_2 NPs	32	16	64	32
THBA-Cu-TOB_3 NPs	8	4	8	8
THBA-Cu-TOB_4 NPs	16	8	32	16

MIC: Minimum Inhibitory Concentration; MBC: Minimum Bactericidal Concentration.

Table S4. Clinical scoring standard.

Structure	Score	Clinical Feature
Conjunctiva	0	normal
	1	mild edema
	2	moderate edema, mild to moderate hyperemia, slight infiltration
	3	severe edema, severe congestion, severe infiltration
Cornea	0	clearness
	1	mild opacity, pupil visible
	2	mild opacity and cover the pupils
	3	moderate opacity, pupil visible
	4	moderate opacity and cover the pupils
	5	severe opacity
	6	corneal ulcer or perforation
Atria	0	normal
	1	a small amount of floating, a small amount of flocculent exudate
	2	mass fiber exudation
	3	empyema in anterior chamber
Iris	0	normal
	1	mild hyperemia
	2	moderate hyperemia
	3	severe hyperemia, adhesion, irregular pupils

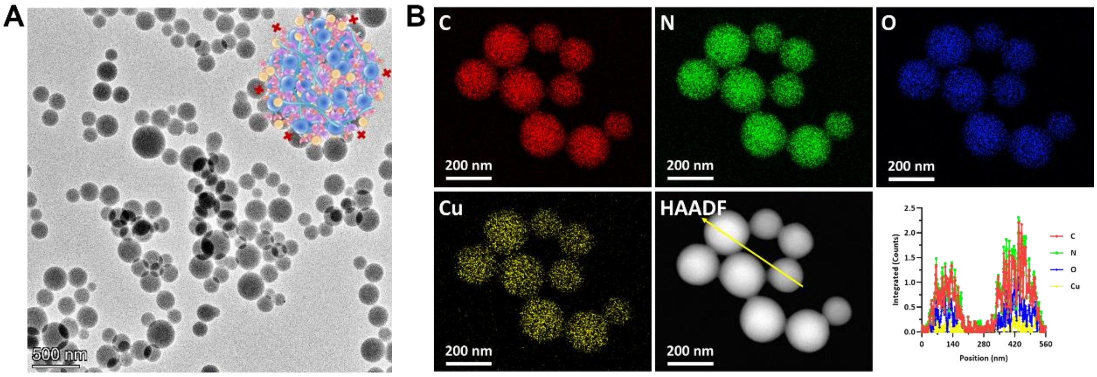

Figure S1. A) TEM and B) corresponding element (C, N, O, and Cu) mapping images for the THBA-Cu NPs. Inside A: schematic representation of the different morphology of NPs (upper-right corner) and photographs of NPs solution with the Tyndall effect (bottom-right-corner). Inside B, line-scan STEM elemental distribution (yellow arrow) of the THBA-Cu NPs.

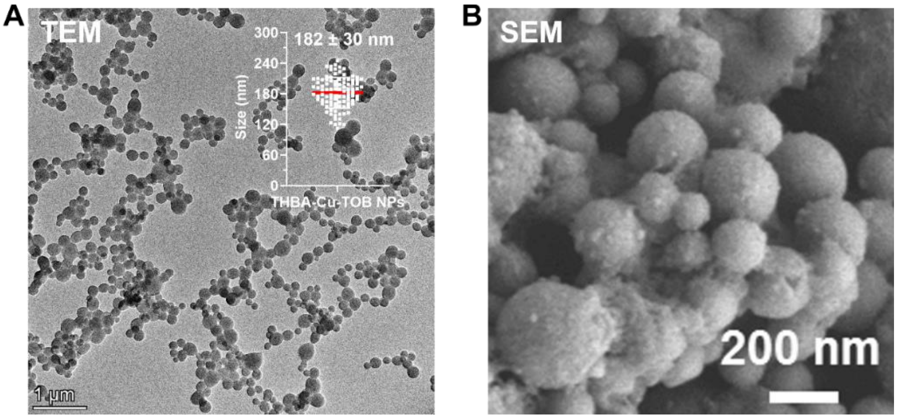

Figure S2. A) TEM and B) SEM images of the THBA-Cu-TOB NPs. Inset: the size of the THBA-Cu-TOB NPs calculated by Image J software.

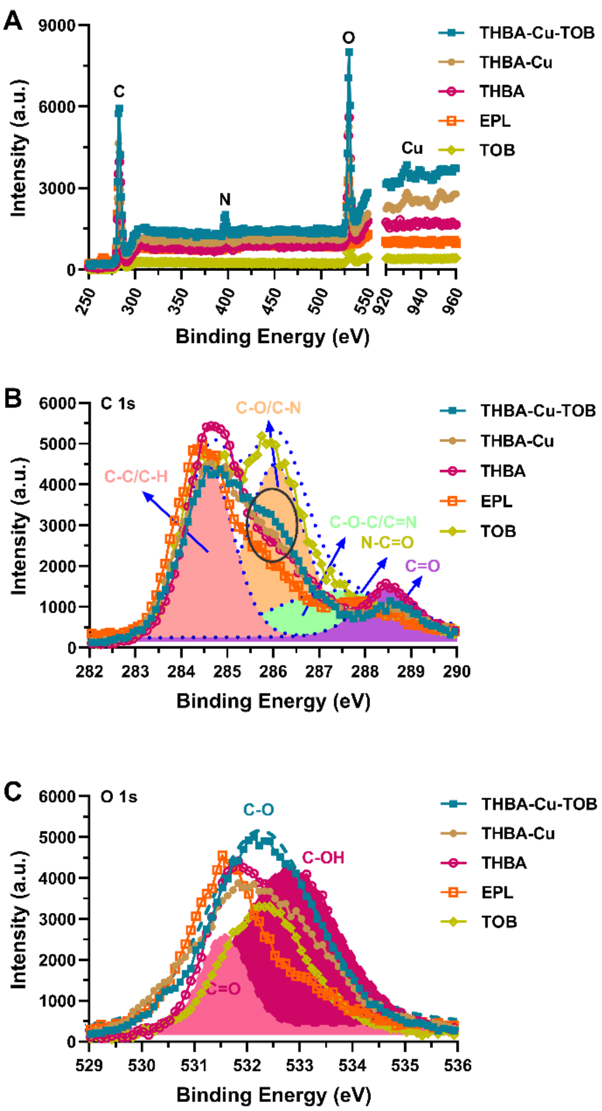

Figure S3. A) Full XPS spectra of the THBA, EPL, TOB, THBA-Cu, and THBA-Cu-TOB. High-resolution XPS spectra for B) C 1s and C) O 1s of the THBA, EPL, TOB, THBA-Cu, and THBA-Cu-TOB, respectively.

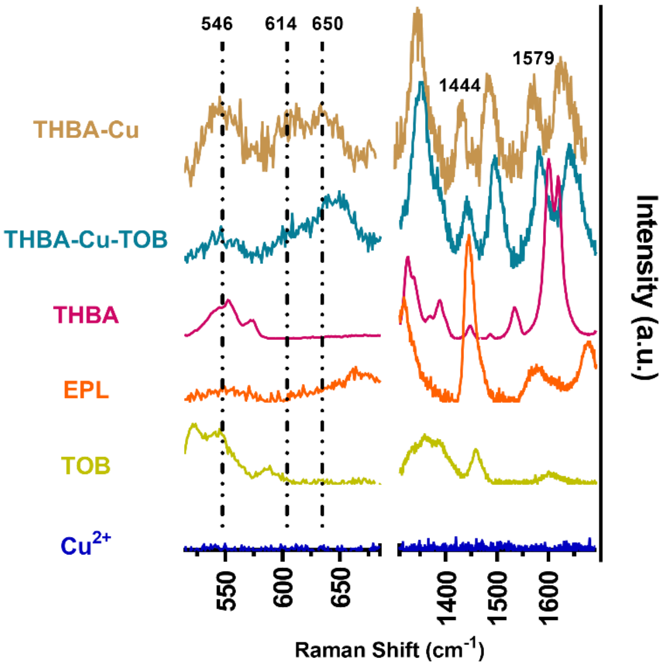

Figure S4. Raman spectrum of the THBA-Cu and THBA-Cu-TOB NPs.

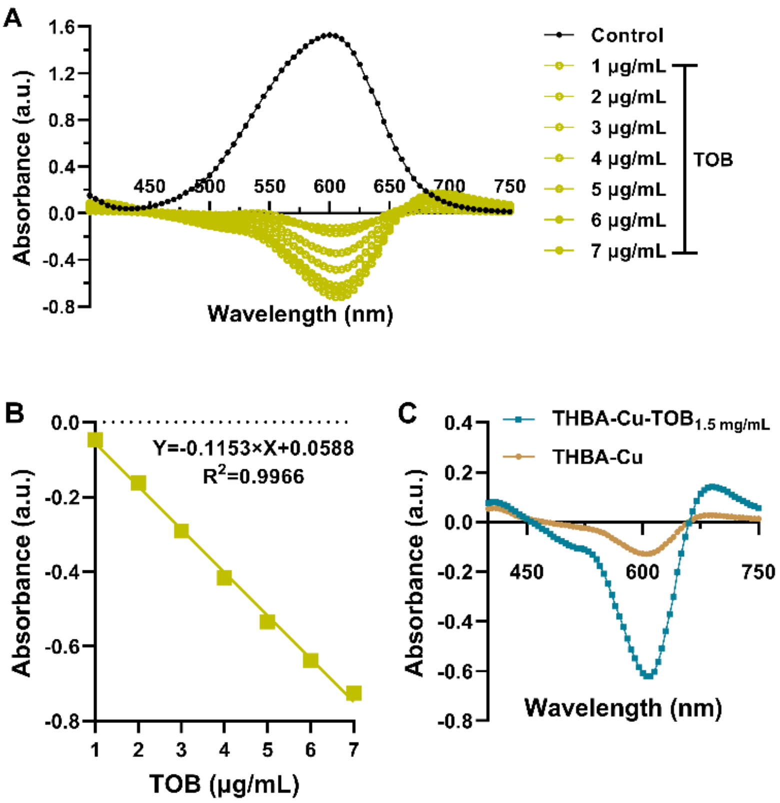

Figure S5. Determination of TOB in NPs by fading spectrophotometric method with aniline blue aqueous solution. A) The UV-vis spectra of TOB at various concentrations. B) The standard curve of UV-vis absorbance at 608 nm to TOB concentration. C) The UV-vis absorbance curve of THBA-Cu and THBA-Cu-TOB.

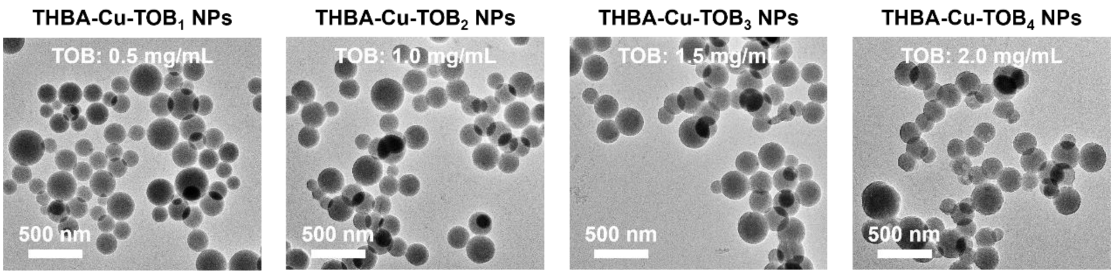

Figure S6. TEM images of the THBA-Cu-TOB NPs with various concentrations of TOB (0.5, 1.0, 1.5, and 2.0 mg/mL).

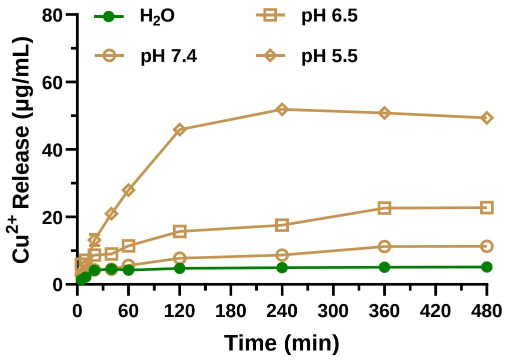

Figure S7. Quantification of Cu^{2+} released from THBA-Cu NPs in PBS solution with different pH determined by ICP-MS.

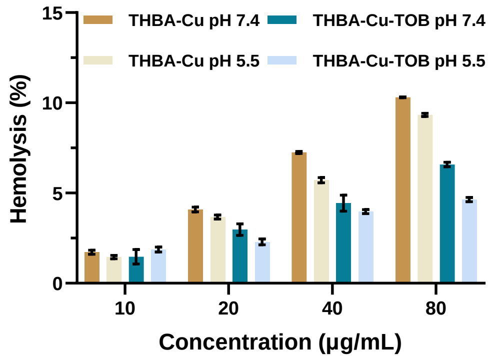

Figure S8. Hemolysis of red blood cells after incubation with NPs with different concentrations at pH 7.4 and 5.5.

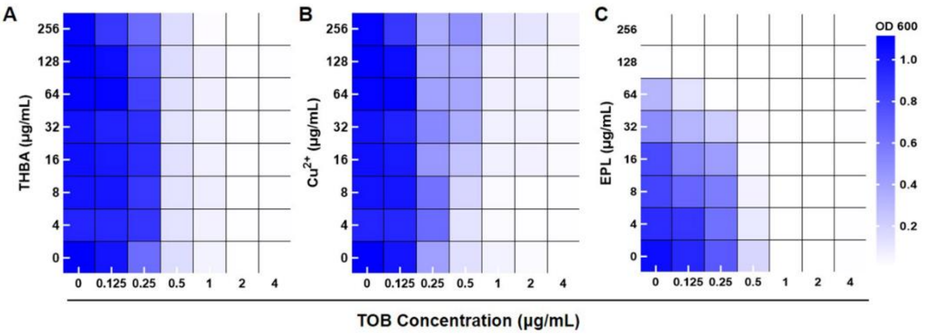

Figure S9. Checkerboard dilution results performed on *P. aeruginosa* to evaluate the synergy between the TOB and A) THBA, B) Cu^{2+}, and C) EPL, respectively.

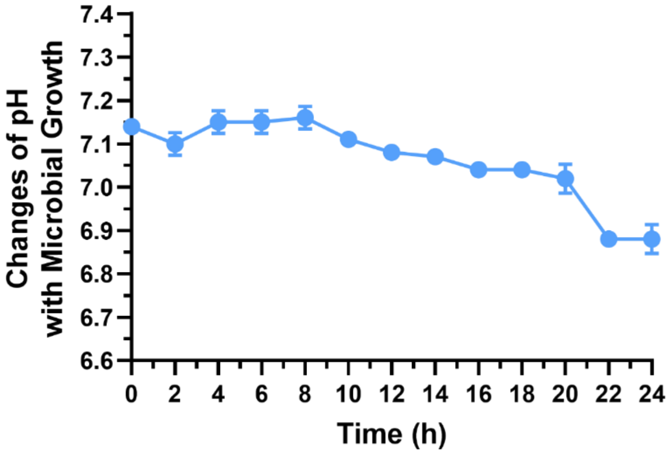

Figure S10. Changes of pH as *P. aeruginosa* growth in TSB solution.

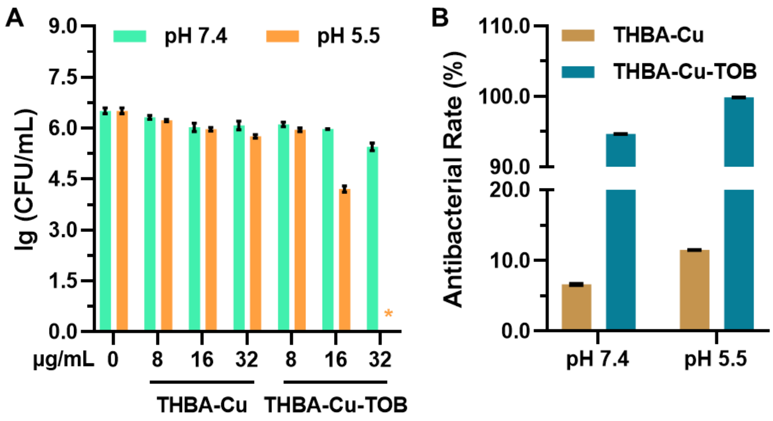

Figure S11. A) Colony-forming units (lg CFU/mL) of *P. aeruginosa* after incubation with different concentrations of NPs at different pH values for 2 h. B) Antibacterial rate of THBA-Cu NPs (32 μg/mL) and THBA-Cu-TOB NPs (32 μg/mL) after 2 h.

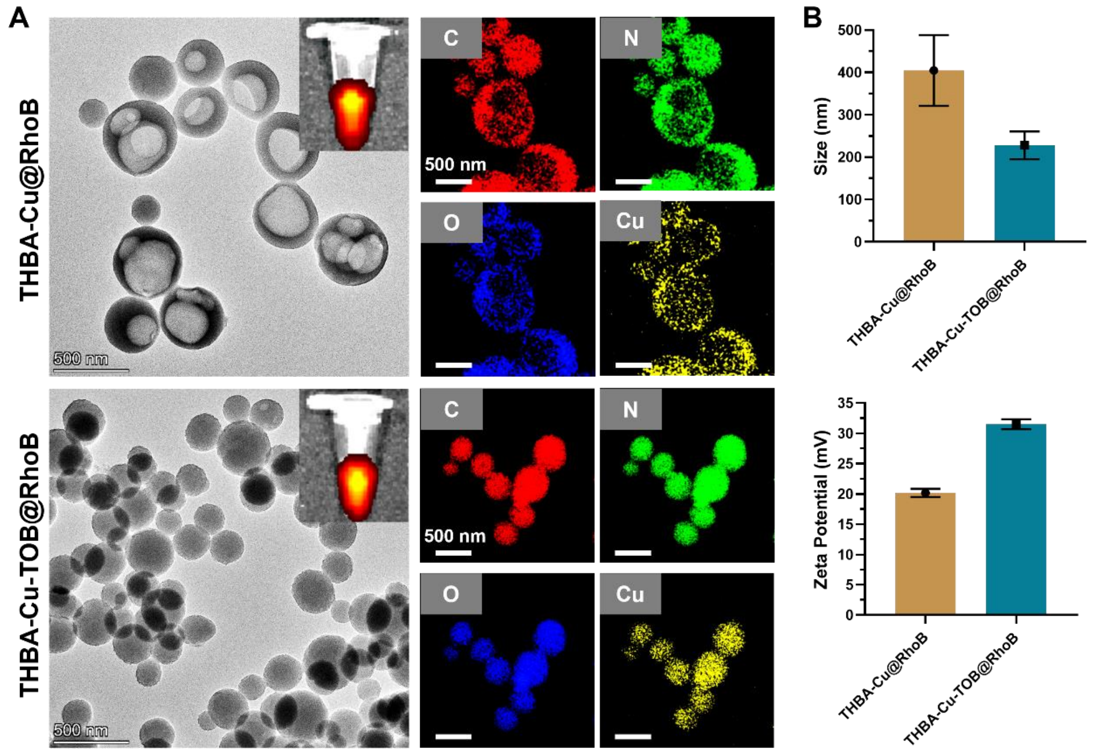

Figure S12. A) TEM images of the morphology of RhoB-labeled THBA-Cu and THBA-Cu-TOB NPs (upper-right corner: fluorescence images of sample solution), and the corresponding element mapping images of THBA-Cu@RhoB and THBA-Cu-TOB@RhoB (C, N, O, and Cu). B) Size and zeta potential of THBA-Cu@RhoB and THBA-Cu-TOB@RhoB.

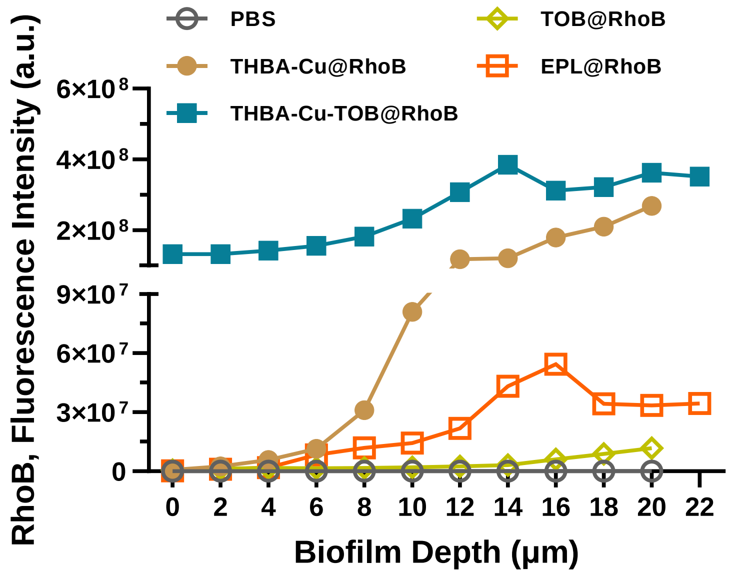

Figure S13. Penetration of THBA-Cu@RhoB, THBA-Cu-TOB@RhoB, TOB@RhoB, and EPL@RhoB into *P. aeruginosa* biofilm through measuring the fluorescence intensity (RhoB).

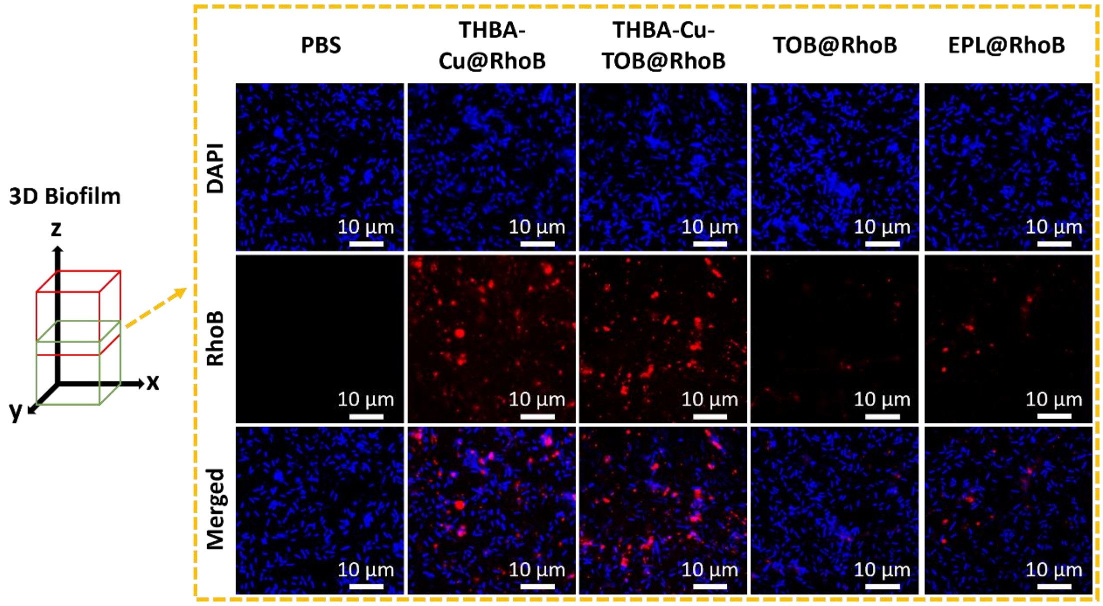

Figure S14. CLSM analysis of the penetration of THBA-Cu@RhoB, THBA-Cu-TOB@RhoB, TOB@RhoB, and EPL@RhoB into *P. aeruginosa* biofilm (DAPI: blue; RhoB: red).

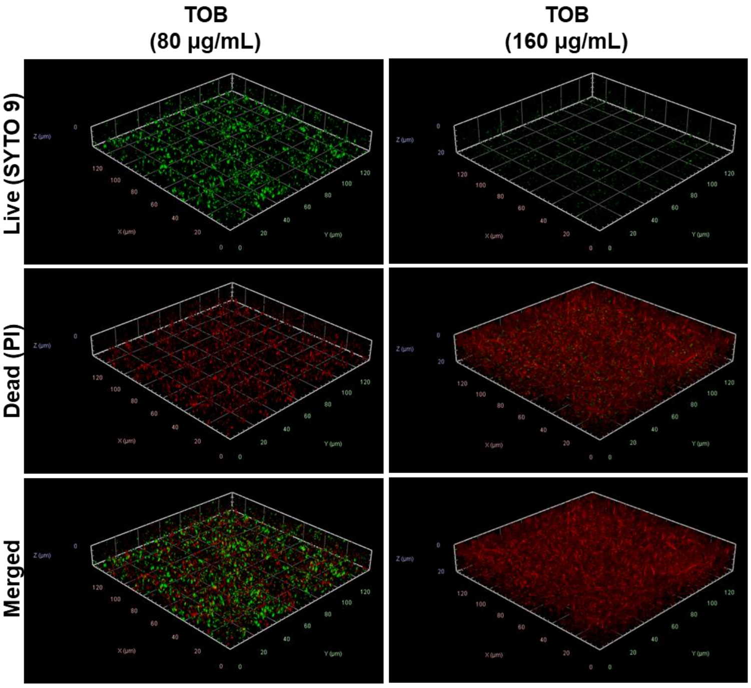

Figure S15. CLSM images of the *P. aeruginosa* biofilm incubated with free TOB (Green: live bacteria; Red: dead bacteria).

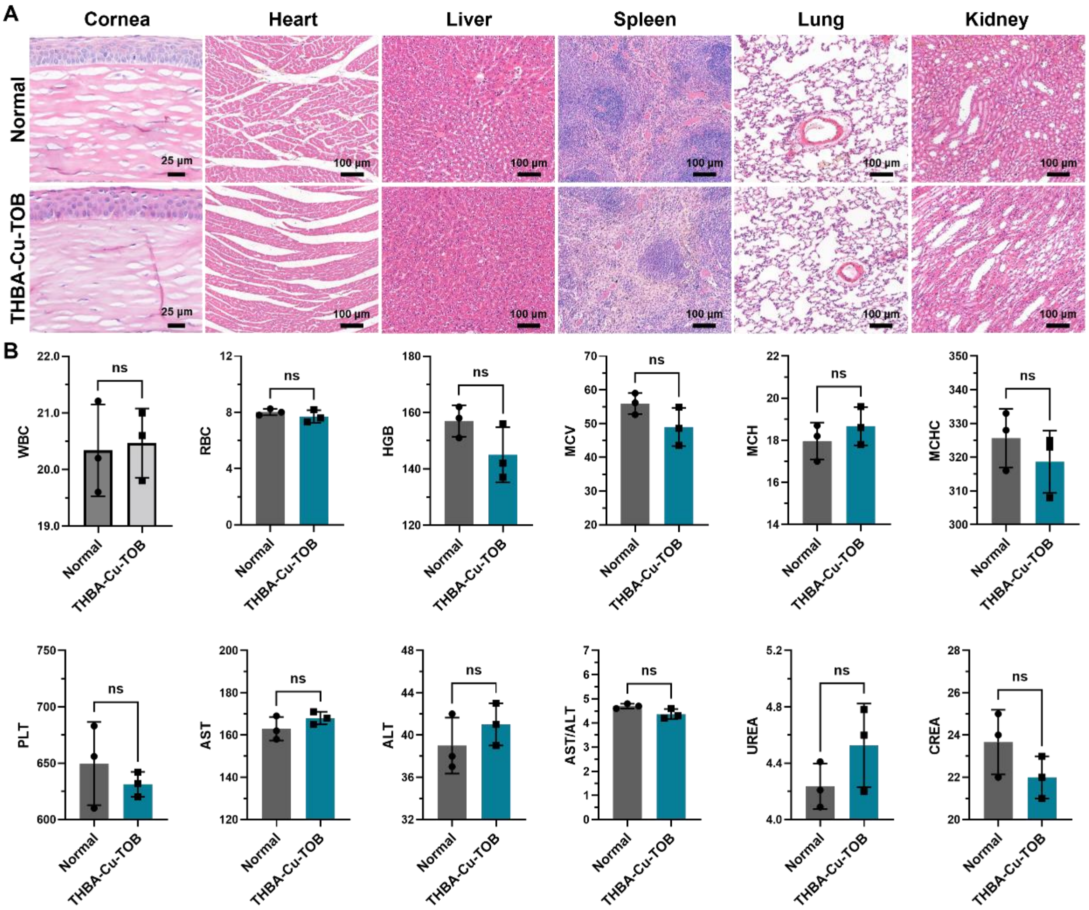

Figure S16. In vivo safety evaluation of THBA-Cu-TOB NPs. A) H&E staining images of cornea, heart, liver, spleen, lung, and kidney in rats. B) Detection of blood biochemical indexes in rats. Data were presented as means ± SD (n = 3): no significant (ns), $p > 0.05$.

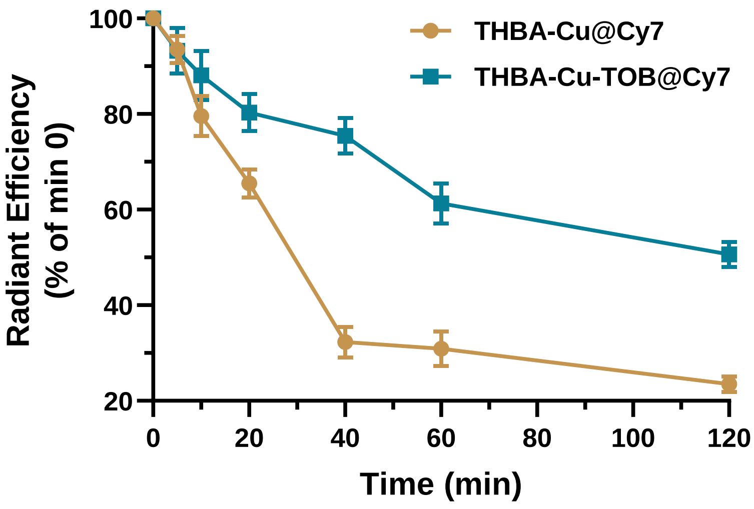

Figure S17. The trend of fluorescence intensity to measure the retention of THBA-Cu and THBA-Cu-TOB NPs on ocular surface.

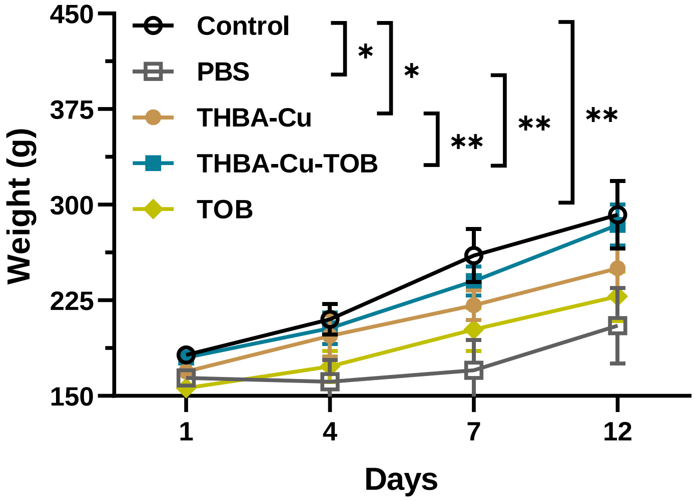

Figure S18. Body weight changes in different groups. Data were presented as means ± SD (n = 5): *$p < 0.05$ and **$p < 0.01$.

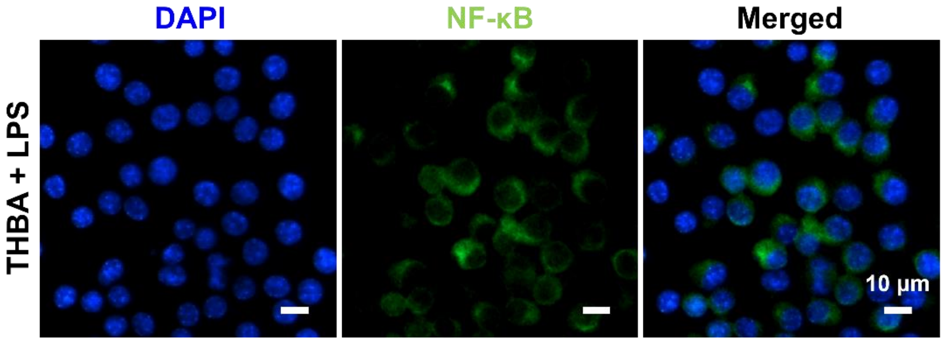

Figure S19. Fluorescence images of RAW264.7 cells treated by THBA. Blue: DAPI; NF-κB: green.

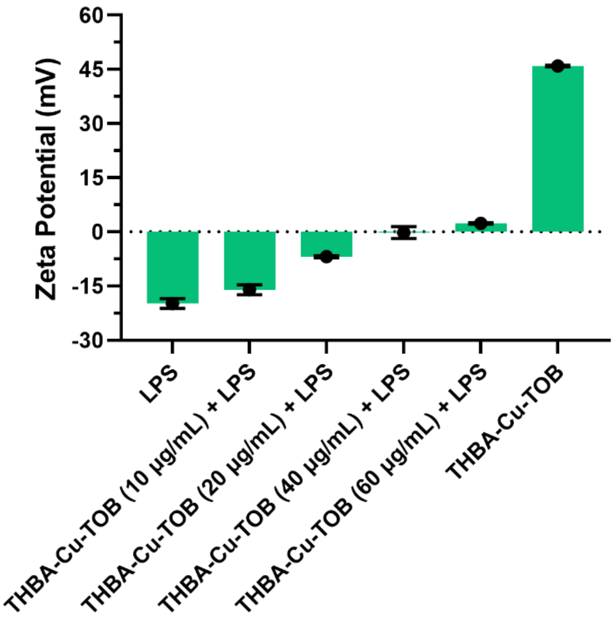

Figure S20. Zeta potential of LPS (5 μg/mL) and LPS-treated THBA-Cu-TOB NPs.

Supplementary References

[S1] S. J. Marrink, H. J. Risselada, S. Yefimov, D. P. Tieleman, A. H. de Vries, *J. Phys. Chem. B* **2007**, 111, 7812.

[S2] S. Li, Z. Lu, Y. Huang, Y. Wang, Q. Jin, X. Shentu, J. Ye, J. Ji, K. Yao, H. Han. *Adv. Sci.* **2022**, 9, 2200435.

[S3] J. Deng, D. Lin, X. Ding, Y. Wang, Y. Hu, H. Shi, L. Chen, B. Chu, L. Lei, C. Wen, J. Wang, Z. Qian, X. Li. *Adv. Funct. Mater.* **2022**, 32, 2109173.

[S4] C. R. Thorn, C. de S. Carvalho-Wodarz, J. C. Horstmann, C. M. Lehr, C. A. Prestidge, N. Thomas. *Small* **2021**, 17, 2100531.
